# Phytochemical characterization of *Typha domingensis* and the assessment of therapeutic potential using *in vitro* and *in vivo* biological activities and *in silico* studies

**DOI:** 10.3389/fchem.2023.1273191

**Published:** 2023-11-08

**Authors:** Rizwana Dilshad, Kashif-ur-Rehman Khan, Saeed Ahmad, Asif Ansari Shaik Mohammad, Asmaa E. Sherif, Huma Rao, Maqsood Ahmad, Bilal Ahmad Ghalloo, M. Yasmin Begum

**Affiliations:** ^1^ Department of Pharmaceutical Chemistry, Faculty of Pharmacy, The Islamia University of Bahawalpur, Bahawalpur, Pakistan; ^2^ Department of Clinical Pharmacy, College of Pharmacy, King Khalid University, Abha, Saudi Arabia; ^3^ Department of Pharmacognosy, College of Pharmacy, Prince Sattam bin Abdul Aziz, Al-Khar, Saudi Arabia; ^4^ Department of Pharmacognosy, Faculty of Pharmacy, Mansoura University, Mansoura, Egypt; ^5^ Department of Pharmaceutics, College of Pharmacy, King Khalid University, Abha, Saudi Arabia

**Keywords:** *T. domingensis* extract, phytochemical profiling by UHPLC–MS, phenolic quantification by HPLC, *in vitro* biological activities, *in vivo* anti-inflammatory and analgesic, *in silico* docking and ADMET predictions

## Abstract

*Typha domingensis*, a medicinal plant with significant traditional importance for curing various human diseases, has potentially bioactive compounds but was less explored previously. Therefore, this study aims to investigate the therapeutic potential of *T. domingensis* by evaluating the phytochemical profile through high-performance liquid chromatography (HPLC) techniques and its biological activities (*in vitro* and *in vivo*) from the methanolic extract derived from the entire plant (TDME). The secondary metabolite profile of TDME regulated by reverse phase ultra-high-performance liquid chromatography–mass spectrometry (RP-UHPLC–MS) revealed some bioactive compounds by -ve and +ve modes of ionization. The HPLC quantification study showed the precise quantity of polyphenols (*p*-coumaric acid, 207.47; gallic acid, 96.25; and kaempferol, 95.78 μg/g extract). The enzyme inhibition assays revealed the IC_50_ of TDME as 44.75 ± 0.51, 52.71 ± 0.01, and 67.19 ± 0.68 µgmL^-1^, which were significant compared to their respective standards (indomethacin, 18.03 ± 0.12; quercetin, 4.11 ± 0.01; and thiourea, 8.97 ± 0.11) for lipoxygenase, α-glucosidase, and urease, respectively. Safety was assessed by *in vitro* hemolysis (4.25% ± 0.16% compared to triton × 100, 93.51% ± 0.36%), which was further confirmed (up to 10 g/kg) by an *in vivo* model of rats. TDME demonstrated significant (*p* < 0.05) potential in analgesic activity by hot plate and tail immersion tests and anti-inflammatory activity by the carrageenan-induced hind paw edema model. Pain latency decreased significantly, and the anti-inflammatory effect increased in a dose-dependent way. Additionally, *in silico* molecular docking revealed that 1,3,4,5-tetracaffeoylquinic acid and formononetin 7-O-glucoside-6″-O-malonate possibly contribute to enzyme inhibitory activities due to their higher binding affinities compared to standard inhibitors. An *in silico* absorption, distribution, metabolism, excretion, and toxicological study also predicted the pharmacokinetics and safety of the chosen compounds identified from TDME. To sum up, it was shown that TDME contains bioactive chemicals and has strong biological activities. The current investigations on *T. domingensis* could be extended to explore its potential applications in nutraceutical industries and encourage the isolation of novel molecules with anti-inflammatory and analgesic effects.

## 1 Introduction

Natural products and their pharmaceutical compounds are used to treat a wide range of human illnesses. Despite advances in medical science over the last few decades, many major diseases remain difficult to cure (Singh, 2010). Medicinal plants have a wide range of bioactive qualities and have been used for therapeutic purposes since ancient times, and they continue to play an important role in modern medical study and practice (Sofowora et al., 2013). Cancer, bacterial infections, and immunological abnormalities are examples of these ailments, and over 25% of all prescribed medications in the world come from plants (Uddin et al., 2011). Chronic inflammatory diseases continue to be a major global health concern and are linked to many health conditions (Khan N. U. et al., 2022). The body’s natural and reversible defense mechanism against a variety of harmful substances, including toxins, microbial attacks, physical agents, and immunological responses, is inflammation. Inflammation that lasts too long or becomes uncontrolled can lead to serious illnesses (Chen et al., 2018; Khan K. et al., 2022).

The naturally occurring bioactive compounds including various phenolics and flavonoids occurring in medicinal plants were found to be promising inhibitors of several enzymes involved in human pathologies (Shahzad et al., 2022). Pain is a sensual modality that, under many circumstances, is the solitary indication used to diagnose a variety of disorders. Throughout history, numerous therapeutic methods have been used for pain relief, with the widespread use of medicinal plants standing out prominently (Uritu et al., 2018). Furthermore, in recent times, there has been a notable increase in the exploration of wild medicinal plants for their potential antidiabetic effects, and it was found that highly effective natural antidiabetic agents that were reported with phytochemicals exhibit several possible modes of action (Ahmed et al., 2022b). Urease inhibitors are also gaining importance due to their potential applications in gastric and urinary tract infections (UTIs). Gastric ulcers, urolithiasis, and UTIs are frequent ailments of this era that involve urease enzymes in their pathology (Nile et al., 2017).

*Typha domingensis*, commonly known as cattail, is a herbaceous, perennial wetland plant found all around the world (Beare and Zedler, 1987). There are 30 species of *Typha* in the family Typhaceae (Pandey and Verma, 2018). Turkish folk medicine uses female *Typha* species topically to stop bleeding (Akkol et al., 2011). Additionally, these were also used for healing wounds and burns. The pollen is astringent, diuretic, desiccant, hemostatic, and vulnerable. The leaves are diuretics. *Typha domingensis* also has nutritional value. Species of *Typha* such as *Typha elephantine* (Roxb), *Typha angustifolia*** (Watt), and *Typha latifolia* (Edgew) are recognized as reservoirs of antimycobacterial agents (Rao et al., 2016). All parts of the *Typha* plant, predominantly the rhizomes, are edible for consumption. The presence of starch grains in grinding stones suggests that they were utilized as a food source in Europe a considerable time ago (Zeng et al., 2020). Because of their higher carbohydrate content than potatoes, rootstocks and rhizomes are consumed as a nutritious food source during the spring season. Furthermore, they have protein levels equivalent to maize and rice (Morton, 1975; Zeng et al., 2020). The root can likewise be dried, ground into a powder, and afterward added to cereal flours or utilized as a thickener in different dishes. This flour, which is high in protein, is used to make bread, cakes, biscuits, etc. (Aljazy et al., 2021).

The goal of this study is to investigate *T. domingensis*, as a medicinal plant, with a variety of useful features, including toxicology and central analgesic activity, as well as anti-inflammatory and enzymatic inhibition activities. This might open the door to the discovery of bioactive chemical substances without the negative side effects usually connected with synthetic drugs.

## 2 Materials and methods

### 2.1 Collection, identification, and preparation of the methanolic extract of *T. domingensis*

The mature plant as a whole was inspected and authorized by the herbarium of the Department of Botany, which is part of the Faculty of Life Science at the Islamia University of Bahawalpur in Pakistan. In March 2019, a specimen was then assigned the reference number 412 and cataloged in the herbarium.

After being collected, the plant was carefully cleaned before being spread out on a fresh sheet of paper to dry naturally for 20 days. The plant pieces were mechanically crushed into a powdery state after drying. Approximately 3 kg of the powdered substance was submerged in 80% methanol for 14 days while being stirred on occasion. To assure further purification, the subsequent solutions were first filtered through a muslin cloth and then through Whatman 1 filter paper having a pore size of 11 µm. By applying reduced pressure to a rotary evaporator (Heidolph Laborota 4,000, Schwabach, Germany), the filtrate that resulted was concentrated into a partially solid mass. A lyophilizer was then used to completely dry the concentrated solution. The semisolid mass was subsequently consolidated in the open air, producing 270 g of the finished product. The labeled *T. domingensis* specimens were properly preserved in an airtight container and set aside for future examination according to the previously reported procedures (Nazir et al., 2021).

### 2.2 Phytochemical analysis of TDME

#### 2.2.1 Reverse phase ultra-high-performance liquid chromatography–mass spectrometry analysis of TDME

The Agilent 1,290 Infinity UHPLC system, which was coupled to the Agilent 6,520 Accurate-Mass Q-TOF mass spectrometer, was used in conjunction with the Agilent Eclipse XDB-C18 column, which had dimensions of 2.1 × 150 mm and a particle size of 3.5 m. Throughout the analysis, the column temperature was held constant at 25 °C, while the auto-sampler temperature was set and held at 4 °C [18]. Mobile phase A, which flowed at a rate of 0.5 mL/min and contained a 0.1% formic acid solution in water, was chosen for the analysis. This was chosen in preference to another mobile phase (mobile phase B), which was a 0.1% solution of formic acid in acetonitrile. The analysis was performed for 25 min after infusing 1.0 µL of plant extracts that had been diluted in methanol of high-performance liquid chromatography (HPLC) grade. A post-run time of 5 minutes was noticed after the run. Nitrogen gas was used to create nebulization at a start-up flow rate of 25 L per hour. This was followed by increasing the flow rate to 600 L per hour to facilitate drying at 350 °C. The fragmentation voltage was kept at 125 V, while the capillary voltage was set at 3500 V (Saleem et al., 2019). Similar to previous findings, RP-UHPLC–MS analyses were carried out to evaluate the polyphenolic composition of TDME. Agilent MassHunter Qualitative Analysis B.05.00 software was used to evaluate the data using the Metabolomics-2017–00004. m method. Utilizing particular criteria, such as a match tolerance of 5 ppm, the compounds were found using the exploration database METLIN_AM_PCDL-Ne 170502 cdb. Both negative ion modes like H- and positive ion modes like H+, Na+, and NH4+ were employed (Locatelli et al., 2017).

#### 2.2.2 Polyphenolic profile by HPLC (quantitative analysis)

**FIGURE 1:**
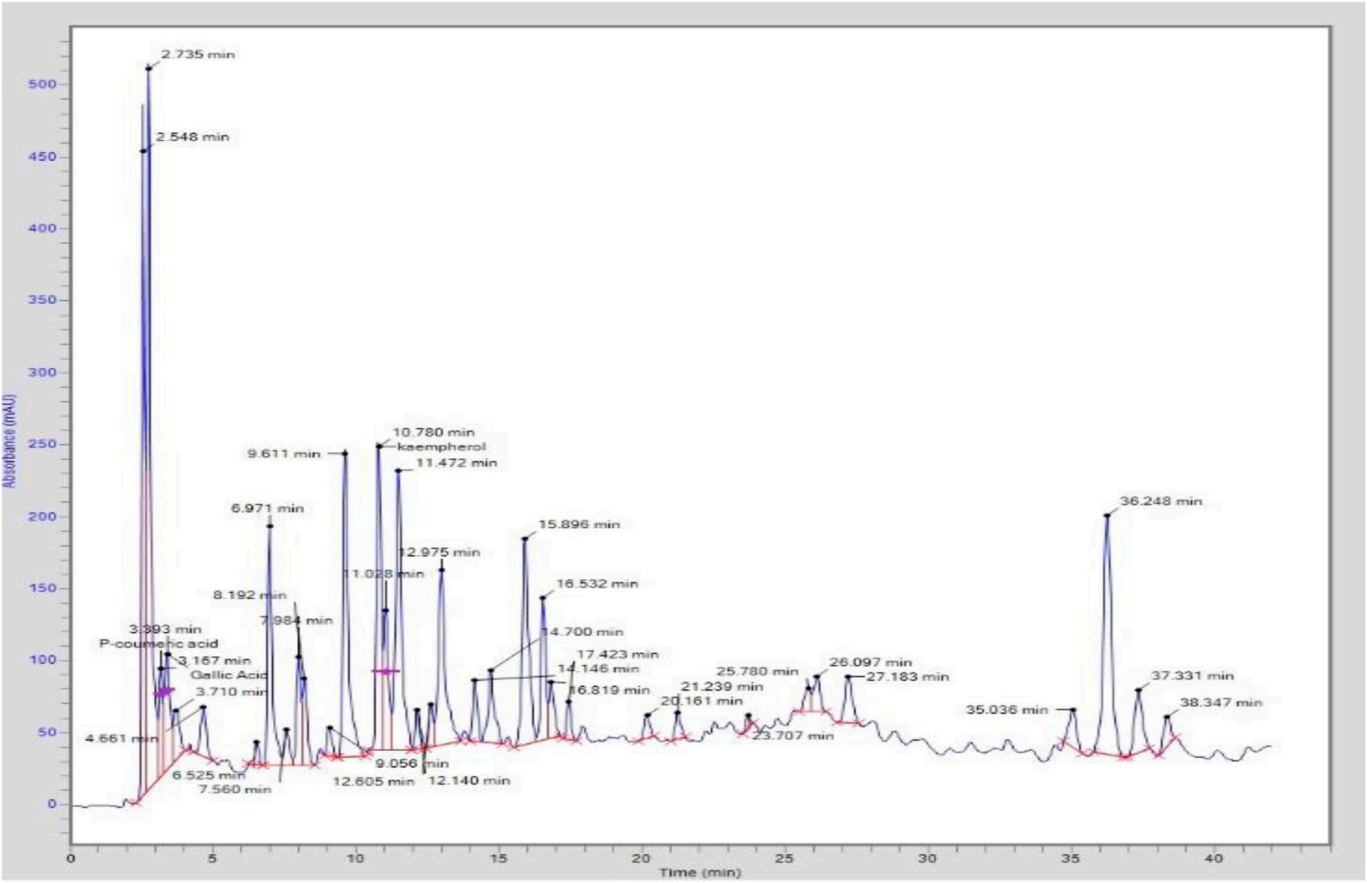
Chromatogram of the phenolic compound profile by HPLC (quantitative analysis).

A Shimadzu HPLC system from Japan was used to quantify the sample extract (Al-Qahtani et al., 2023). To determine the phenolic profile, a dried sample weighing 0.1 g was combined with 1 mL of methanol. The sample was filtered through a 0.45 μm syringe filter and centrifuged at 6,000 *g* for 15 min. A 10 µL volume was then injected into the HPLC apparatus after that. The Shimadzu Shim-pack CLC-ODS C-18 column (with dimensions of 5 cm × 4.5 mm and a particle size of 5 m) was used to analyze the samples. The standards used in this analysis for the quantification of polyphenols are chlorogenic acid, *p*-coumaric acid, gallic acid, ƿ-hyydroxy benzoic acid, caffeic acid, vanillic acid, kaempferol, sinapic acid, ferulic acid, salicylic acid, coumarin, quercetin, benzoic acid, and rutin. The calculation of the k-factor was used. The limit of detection and limit of quantification of these compounds were in the range of 0.15–0.46 and 0.42–2.47 μg/mL, respectively. Glacial acetic acid (1% in water), which was solution A, and methanol B were combined in the mobile phase at the volumetric ratios of 90:10, 84:16, 72:28, 65:35, 50:50, and finally, to 90:10 (acetic acid:methanol) at time intervals of 0–5, 6–20, 21–35, 36–45, and 46–60 min, and the final concentration in the mobile phase was run for 61–75 min to restore the original conditions, before the new sample was injected. The flow rate was maintained at 0.8 mL/min, and the injection volume was 10 µL. An RS Diode UV–VIS matrix detector was used to detect the signals of chromatogram and operated in a wavelength range of 270–370 nm. The temperature of the column was maintained at 25 °C, and identification of peaks was performed by the comparison of peaks obtained by running the standard compounds under similar conditions. The similar procedure was also reported by Basit et al. (2023). The experiment was performed in triplicates, and the obtained chromatogram is shown in Figure 1.

$$\text{Concentration of compound }\left(µg/g\right)=\text{Area of the Peak }*\;k‐\text{factor of the compound}.$$

### 2.3 *In vitro* biological investigation

#### 2.3.1 Enzyme inhibition assays

##### 2.3.1.1 Lipoxygenase inhibition activity

The anti-lipoxygenase action was evaluated using the same modification test techniques as previously published (Singsai et al., 2020). Lipoxygenase from soybean and linoleic acid (substrate) were used in their native forms as supplied by Sigma-Aldrich, and the extract’s analytic solution was prepared in methanol (1 mg/mL) and then diluted in different aliquots (0.05–1 mg/mL). Following the procedure indicated by Singsai et al., the reaction solution was created, and then, 1.0 mL of substrate was added (linoleic acid at a concentration of 0.6 mM) following the incubation process. At 234 nm, the absorbance was measured after thorough mixing. As a reference inhibitor of lipoxygenase, indomethacin at the concentration of 1 mg/mL in methanol (with dilutions as mentioned for the extract) was used to quantify the percentage of inhibition. The percentage of inhibition was calculated by comparing it to the inhibitory action of indomethacin, a well-known standard lipoxygenase inhibitor. Then, the percentage inhibition was determined using the following expression:

$$\text{Lipoxygenase enzyme inhibition }\left(\%\right)=\frac{\left(\text{Absorb}.\;\text{of}\;\text{sample}-\text{Absorb}.\;\text{of}\;\text{negative}\;\text{control}\right)}{\text{Absorb}.\;\text{of}\;\text{positive}\;\text{control}}\times 100.$$

##### 2.3.1.2 α-Glucosidase inhibition activity

The standard procedure was already established (Nisar et al., 2022) for the α-glucosidase inhibition activity, with a few minor adjustments. The extracts and the standard solutions were prepared in the concentration of 1 mg/mL along with different dilutions of 0.05–1 mg/mL. An α-glucosidase enzyme solution of 1U/mL in 50 mM phosphate buffer (pH 6.8), 0.5 mM para-nitrophenyl-β-D-glucopyranoside (pNPG) solution, and standard solution or positive control (acarbose) with different concentrations was prepared. The α-glucosidase enzyme (10 μL) was added to 96-well plates for the α-glucosidase inhibition experiment. The wells were formerly filled with 20 µL of the extracts or a reference solution and 50 µL of phosphate buffer at a pH of 6.8. After 15 min of incubation at 37 °C to stop the enzymatic activity, the mixture was tested for absorbance at a wavelength of 405 nm. The same response mixture received 20 µL of a 0.5 mM solution of para-nitrophenyl-D-glucopyranoside (pNPG). The mixture was allowed to sit at room temperature for 30 min. Using the provided formula, the final absorbance at 405 nm was measured to estimate the level of inhibition of the α-glucosidase enzyme. Methanol (20 μL) was added as the blank in place of the sample/standard.

$$\alpha -\text{Glucosidase enzyme inhibition }\left(\%\right)=\frac{\left(\text{Absorb}.\;\text{of}\;\text{sample}-\text{Absorb}.\;\text{of}\;\text{negative}\;\text{control}\right)}{\text{Absorb}.\;\text{of}\;\text{positive}\;\text{control}}\times 100.$$

##### 2.3.1.3 Urease inhibition activity

The urease inhibition assay for TDME was appropriate with minor changes to an earlier described procedure (Nisar et al., 2022). The extract and standard (thiourea) solutions were prepared at a concentration of 1 mg/mL, and different dilutions were also prepared (0.05–1 mg/mL). In 1M phosphate buffer with a pH of 7.0, the urease enzyme was produced as a solution at a concentration of 0.025%. The produced enzyme solution was then poured (20 µL) into 96-well plates. Methanol (20 μL) was added as the blank in place of the sample/standard. The reaction mixture was then given a 60 µL addition of a 2.25% urea solution after the preceding procedures. The resulting mixture was then incubated for 15 min at a temperature of 37 °C. The mixture’s absorbance was then assessed at λ:630 nm. The reaction mixture was then given a 60 µL boost from the phenol reagent before receiving a 100 µL boost from the sodium hypochlorite solution. The resulting mixture was then incubated at a temperature of 37 °C for further 15 min. The reaction mixture’s absorbance was subsequently measured at a wavelength of 630 nm. The technique for the negative control was also performed as described previously, except that 20 µL of methanol was used in place of the extracts or standards. The following formula determines the inhibition of the urease enzyme. IC_50_ values were used to represent the results.

$$\text{Urease enzyme inhibition }\left(\%\right)=\frac{\left(\text{Absorb}.\;\text{of}\;\text{sample}-\text{Absorb}.\;\text{of}\;\text{negative}\;\text{control}\right)}{\text{Absorb}.\;\text{of}\;\text{positive}\;\text{control}}\times 100.$$

#### 2.3.4 Hemolytic activity

The method outlined earlier was utilized to measure the hemolytic impact of the TDME. A total of 10 mL of human blood was obtained from volunteer participants and placed in a sterile EDTA tube with a screw top. The tube was then put in a centrifuge and spun for 5 min at an acceleration of 850×*g*. After removing the top layer, the erythrocytes were carefully washed with 10 mL of sterile, cooled, isotonic PBS (pH 7.4). The previously cleaned erythrocytes were then reconstituted in 20 mL of sterile, cold PBS. The extracts were then added to the erythrocyte solution at a concentration of 1,000 μg/mL, and the mixture was then incubated at 37 °C for 60 min. The absorbance of hemoglobin in the supernatant at 540 nm was measured to estimate the rate of hemolysis. The proportion of hemolysis was computed using the stated formula. Triton X-100 (0.1%) was used as the +ve control, and PBS was used as the -ve control (Tabassum et al., 2022).

$$\text{Hemolysis percentage }\left(\%\right)=\frac{\left(\text{Absorb}.\;\text{of}\;\text{sample}\;‐\;\text{Absorb}.\;\text{of}\;\text{negative}\;\text{control}\right)}{\text{Absorb}.\;\text{of}\;\text{positive}\;\text{control}}\times 100.$$

#### 2.3.5 *In vitro* anti-inflammatory activity

The technique of stabilizing the membrane of the human red blood cell was exploited in this study. Healthy human volunteers’ blood samples were combined with an equal amount of Alsever’s solution (5 mL blood sample and 5 mL Alsever’s solution). Alsever’s solution consists of 2% dextrose, 0.8% sodium citrate, 0.05% citric acid, and 0.42% sodium chloride. The mixture of blood sample and Alsever’s solution was formerly centrifuged at room temperature for 15 min at a speed of 3,000 rpm. These RBCs were washed with saline solution to eliminate impurities and to prepare a 10% suspension of RBCs. At the time of the experiment, different preparations were made: a control using distilled water and a standard solution of diclofenac sodium (at a conc. of 500 μg/mL and various concentrations of TDME at 500, 1,000, and 3,000 μg/mL). Distilled water, 1 mL of phosphate buffer, 2 mL of hyposaline (0.36%), and last, 0.5 mL of the red blood cell (RBC) suspension were added to each of these preparations in that order. The study followed the guidelines set in the Helsinki Declaration and received approval from the institutional ethical committee known as PREC. The assay combination was centrifuged for 20 min at a speed of 3,000 rpm following a 30-min incubation at 37 °C. At 560 nm, the supernatant solution’s hemoglobin content was calculated by spectrophotometry (Chirumamilla et al., 2022).

$$\%\text{ inhibition}=1\;–\;\left(\text{Absorbance of sample}/\text{ Absorbance of control}\right)*\;100.$$

### 2.4 *In vivo* biological evaluation

#### 2.4.1 Chemicals

All of the materials utilized in the study were highly pure and appropriate for scientific investigation. Sigma-Aldrich (United States) provided carrageenan, Novartis Pharma Ltd. provided diclofenac sodium, and Indus Pharma provided pentazocine (Javed et al., 2020).

#### 2.4.2 Drugs

TDME (at dosages of 30, 100, and 300 mg/kg), pentazocine (at a dose of 10 mg/kg), diclofenac sodium, and indomethacin (at a dose of 15 mg/kg) were produced and dissolved in distilled water. These solutions were given intraperitoneally at a dosage of 5 mL/kg, acting as reference substances for assessing their anti-inflammatory and analgesic effects (Javed et al., 2020).

#### 2.4.3 Experimental animals

The Wistar albino rats were housed and cared for in the animal facility of the Pharmacology & Physiology research laboratory in Pakistan (the Islamia University of Bahawalpur’s Faculty of Pharmacy and Alternative Medicine). The rats were both male and female, weighing between 140 and 225 g. In this investigation, polycarbonate cages with a maximum animal occupancy of six were used, measuring 47 × 34 × 18 cm^3^. Standard environmental parameters were upheld throughout the trial, including a temperature of 25°C ± 2 °C, humidity levels of 50%–55%, and a consistent 12-h cycle of light and darkness. Throughout the trial, the animals received standard animal feed and had full access to water. The experimental circumstances were kept up for a week before the trial began to reduce stress on the animals. Under the reference number PAEC22/74, the institution’s Pharmacy Research Ethics Committee (PREC) authorized the study’s protocols and procedures (Basit et al., 2022).

#### 2.4.4 Acute toxicity assay

An acute toxicity experiment was used to evaluate the safety of the TDME, with a few minor adjustments made based on the previously published research (Lalitha et al., 2012). Five groups of Wistar albino rats, each with five rats, were constructed, having both males and females in each group. The rats were given plenty of food and drink before the trial began, and they also got used to the laboratory environment. The rats that had been fasting for 12 h were given TDME at increasing doses (0.3, 1, 3, and 10 g/kg). The intraperitoneal (i.p.) method was used to administer the extracts. The study also included a control group that received the normal saline solution (10 mL/kg) for comparison. For the first 12 h, the rat was monitored every 1 h and then daily for the next 14 days. The mortality rate was measured after 48 h, and various physical and behavioral signs were identified and closely tracked in the animals. Tremors, convulsions, salivation, perspiration, lacrimation (tear production), the writhing reflex, somatomotor activity, and any notable behavioral abnormalities were among the symptoms.

#### 2.4.5 *In vivo* anti-inflammatory activity

A modified version of a previously established approach was used to examine the anti-inflammatory properties of TDME. For this assessment, the carrageenan-induced paw edema model was used (Basit et al., 2022). Thirty rats were taken. Six rats were included in each of the five groups from which the animals were separated. As a baseline measurement, the right hind paw of each rat was estimated with a screw gauge. The test drug, TDME, was given to separate groups of rats intraperitoneally (i.p.) at dosages of 30, 100, and 300 mg/kg. Distilled water was used as the administration method, and a dose of 5 mL/kg intraperitoneally was applied. Another group received a normal medication, indomethacin, at a dose of 15 mg/kg i. p. After giving the rats these drugs, 0.1 mL of a 1% carrageenan solution was injected into the plantar tissue of their right hind feet to cause edema. Before 1, 2, 3, and 4 h after the injection of carrageenan, the width of the paws was measured. The difference in paw thickness was calculated by deducting the first measurement (“0 h”) from the measurements collected at the following hourly intervals to calculate the growth in paw thickness (Vázquez et al., 1996).

#### 2.4.6 Analgesic activity

The study used six groups of Wistar albino rats weighing between 150 and 200g and consisted of both males and females. Each group had six animals. The control group of rats received 5 mL/kg of distilled water intraperitoneally (i.p.). The control group obtained intraperitoneal (i.p.) injections of 15 mg/kg diclofenac sodium and 10 mg/kg pentazocine. The other groups received TDME intraperitoneally (i.p.) at dosages of 30, 100, and 300 mg/kg (Javed et al., 2020).

##### 2.4.6.1 Hot plate test

The process mentioned previously was carried out with minor adjustments (Sajid-Ur-Rehman et al., 2021). The hot plate test was carried out at a persistent temperature of 52 °C. By examining the rats’ responses to thermal discomfort, such as paw licking or jumping, the latency period, or reaction time, was assessed in seconds. The reaction time was tested before administration (0 min), as well as 30, 60, 90, and 120 min after administration. A maximum duration limit of 25 s was set to prevent any damage to the paws.

##### 2.4.6.2 Tail immersion test

A water heater was used as the instrument for the tail-flick test. The thermostat was adjusted to keep the water bath at a constant temperature of 53°C ± 2 °C. The bottom section of each rat’s tail was measured and plunged in warmed water, which remained at 53°C ± 2 °C. The rats reacted by pulling their tails out of the hot water. The initial latency was measured first, followed by the administration of the proper dosage to each group. The reaction time was then assessed at 30 min, 60 min, 90 min, and 120 min following intravenous injection. To avoid tissue injury, the maximum allowable duration for tail flick measurement was determined at 25 s (Sewell and Spencer, 1976).

### 2.5 *In silico* prediction studies

#### 2.5.1 Molecular docking

Computer-aided drug design and the advancement in molecular biology both benefit from the use of molecular docking. For molecular recovery, a technique for producing compounds as PDB files and a targeted search database in a valid Protein Data Bank (PDB) format is required. For this reason, many technologies such as the AutoDock Vina program, MGL Tools, Discovery Studio, PyRx, and Babel can be used. The PDB was used to find the receptor molecule used for the investigation. The responsibility for additional receptor preparation fell to Discovery Studio (Discovery Studio 2021 client). All the compounds screened by LC–MS were downloaded from PubChem as SDF files (structured data format). To make ligand compounds, the Babel was employed. Then, Vina, which was integrated into PyRx, was given the receptors and ligands. Specific target proteins were selected for the *in vitro* or *in vivo* assays involving them. α-Glucosidase (1obb) and urease (3la4) were used to find the relationship of compounds with *in vitro* inhibition properties of TDME. Lipoxygenase (6ncf) and cyclooxygenase (6y3c.COX1 and 6bl4. COX2) proteins were selected to evaluate the effect of compounds on *in vitro* and *in vivo* anti-inflammatory, analgesic, and antipyretic activities of TDME. The incorporation of tiny protein molecules into a target requires a planned sample of the ligand’s ability to precisely conform to a certain target pattern to produce an ideal complex form. By using the program evaluation function, this may be done. Vina was applied for docking, and Discovery Studio was used to display the findings of interactions from the docking (Meng et al., 2011).

#### 2.5.2 *In silico* absorption, distribution, metabolism, excretion, and toxicological characterization of compounds

On 28 June 2023, the absorption, distribution, metabolism, and excretion (ADME) features of bioactive compounds derived from reverse phase ultra-high-performance liquid chromatography–quadrupole time-of-flight mass spectrometry (RP-UHPLC–QTOF-MS) were evaluated by using SwissADME (https://www.swissadme.ch/) online tools, and these substances also underwent docking testing against several enzymes (Khan et al., 2023). Protox-II (https://tox-new.charite.de/) was used to evaluate the toxicity by following the reported procedure (Abdullahi et al., 2021).

### 2.6 Statistical analysis

The data detailed were given as the mean standard error of the mean (SEM) after three repetitions of each measurement. Following a one-way ANOVA, the LSD *post hoc* test was used for statistical analysis. The significance level of *p* ≤ 0.05 was used. Prism GraphPad-7 software was used to analyze the experimental data.

## 3 Results and discussion

### 3.1 Analysis and identification of secondary metabolites by RP-UHPLC–QTOF-MS

**FIGURE 2:**
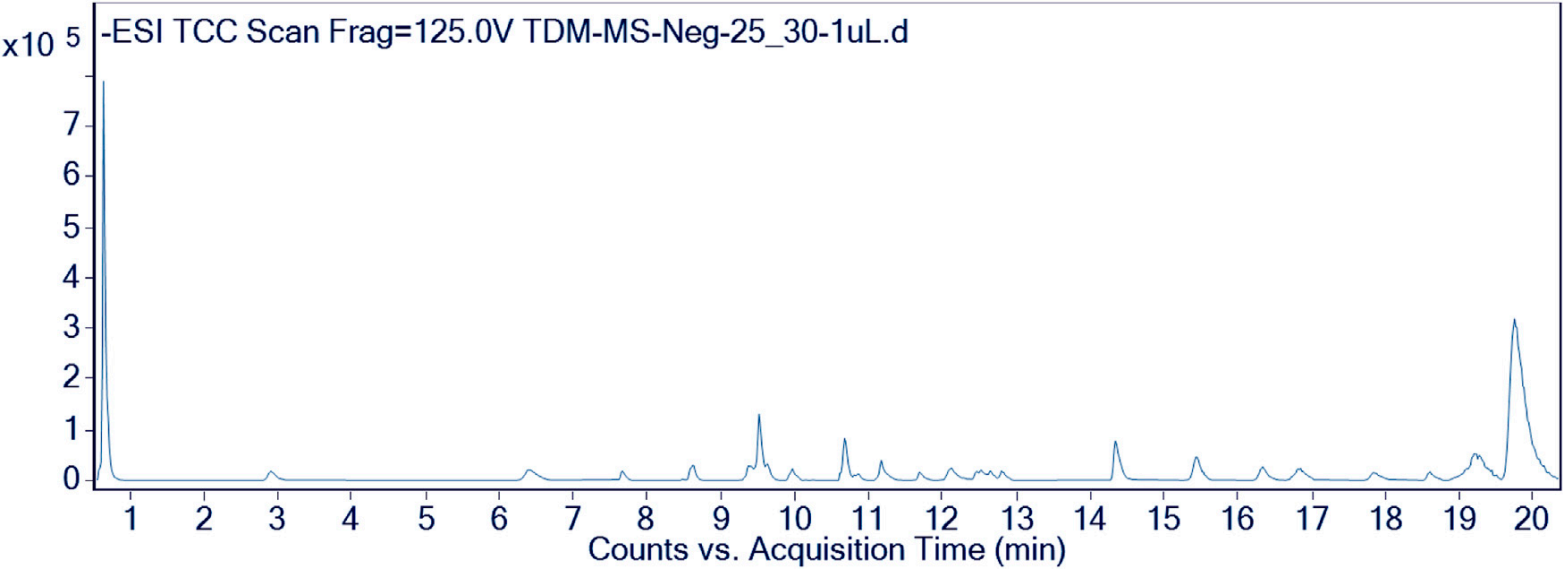
Chromatographic representation of TDME by using UHPLC-Q–TOF-MS (-ve mode).

**FIGURE 3:**
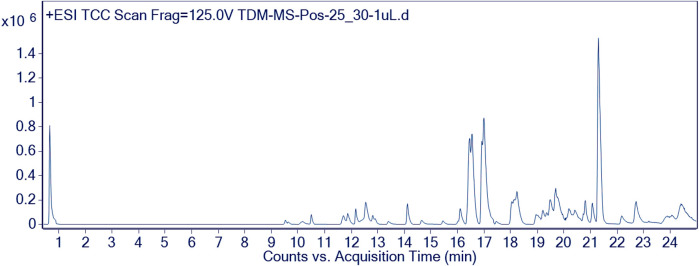
Chromatographic representation of TDME by using UHPLC-Q–TOF-MS (+ve mode).

Furthermore, the methanolic extract of TDME was examined by using reverse phase ultra-high-performance liquid chromatography–mass spectrometry (RP-UHPLC–MS) to acquire comprehensive profiles of several secondary metabolites. To visualize the identified chemicals, standard total ion chromatograms with mass spectrometric peaks for both samples were prepared. This is the first time, according to our research, that this plant has been documented in such detail. Secondary metabolite profiles of TDME were regulated using RP-UHPLC–MS in both -ve and +ve modes. The -ve mode of ionization is shown in Table 1 and Figure 2, as well as Supplementary Figure S1 and Supplementary Figure S2, while the +ve mode is shown in Table 2 and Figure 3, as well as Supplementary Figure S3 and Supplementary Figure S4.

**TABLE 1:** The secondary metabolites from TDME were tentatively identified using UHPLC-Q–TOF-MS analysis in the -ve ionization mode.

RT	M. Mass	Identification	M. Formula	B. Peak (*m/z*)	Chemical class	Reported biological activity
0.64	226.07	5-Acetylamino-6-formylamino-3-methylurssacil	C_8_H_10_N_4_O_4_	225.06	Pyrimidine-dione	Acetyltransferase; Tang et al. (1983)
0.64	180.06	Theobromine	C_7_H_8_N_4_O_2_	179.05	Dimethyl xanthine	Antitussive; Usmani et al. (2005)
6.42	247.99	Vanillic acid 4-sulfate	C_8_H_8_O_7_S	246.99	Phenolic acids	Anti-inflammatory and antidiabetic; Cutler et al. 2018()
9.64	188.10	Nonic acid	C_9_H_16_ O_4_	187.09	Carboxylic acid	
11.18	840.18	1.3,4,5-Tetracaffeoylquinic acid	C_43_H_36_O_18_	839.18	Hydroxycinnamic acid	Anti-inflammatory, analgesic, and antioxidant; Yeboah et al. (2022)
11.70	208	Stipitatonate	C_9_H_4_O_6_	206.99	Stipitatonates	
12.47	222.12	Annuionone B	C_13_H_18_O_3_	221.11	Oxepanes	Allelopathic agents; Macıas et al. (2004)

**RT**, retention time; **M. Mass**, molecular mass; **M. Formula**, molecular formula; **B. Peak**, base peak.

**TABLE 2:** The secondary metabolites from TDME were tentatively identified using UHPLC-Q–TOF-MS analysis in the +ve ionization mode.

RT	M. Mass	Identification	M. Formula	B. Peak (*m/z*)	Chemical class	Reported biological activity
0.65	180.06	L-galactose	C_6_H_12_O_6_	198.09	Aldohexoses	Glactosemia and neurological symptoms; Conte et al. (2021)
0.65	126.03	4-Hydroxy-6-methylpyran-2-one	C_6_H_6_O_3_	127.03	Pyranone	Antimicrobial; Mohamed-Smati et al. (2021)
0.65	162.05	3-Hydroxy-3-methyl-glutaric acid	C_6_H_10_O_5_	180.08	Hydroxy acid	Inhibition of hepatic cholesterol synthesis; Beg and Lupien (1972)
0.65	144.04	Methylitaconate	C_6_H_8_O_4_	145.04	Itaconic acid	
0.66	342.11	Nigerose (Sakebiose)	C_12_H_22_O_11_	360.15	Disaccharide	Anticarcinogenic activity; Konishi and Shindo (1997)
0.67	254.10	Galactosylglycerol	C_9_H_18_O_8_	277.08	Galactolipids	Antitumor; Wei et al. (2013)
0.70	341.13	6-(alpha-D-glucosaminyl)-1D-myo-inositol	C_12_H_23_NO_10_	342.13	Inositol glycon	Antimicrobial and antioxidant; Hamadou et al. (2022)
0.79	120.04	Purine	C_5_H_4_N_4_	121.05	Hetrocyclic aromatic organic compound	Gout and leukemia; Huang et al. (2021)
9.52	516.12	Formononetin 7-O-glucoside-6″-O-malonate	C_25_H_24_O_12_	517.13	Flavonoid glycoside	Anti-inflammatory and antioxidant activity; Hinderer et al. (1987)
11.87	162.06	8Z-decene-4,6-diynoic acid	C_10_H_10_O_2_	163.07	Fatty acid	Antioxidant; Ahmad et al. (2016)
12.17	273.26	C16 sphinganine	C_16_H_35_NO_2_	274.27	Sphingolipids	Schizophrenia; Song et al. (2023)
12.91	267.14	Codonopsine	C_14_H_21_NO_4_	268.15	Pyrrolidizine alkaloid	Antimicrobial activity; El-Nezhawy et al. (2019)
13.41	176.04	4-Methylumbelliferone	C_10_H_8_O_3_	177.05	Coumarin	Anti-cancer activity; Sinha et al. (2019)
14.65	194.09	Ethyl 4-methylphenoxyacetate	C_11_H_14_O_3_	195.10	Phenoxyacetic acid derivative	Flavoring agents; Ahmad et al. (2016)
16.03	234.16	Curcumenol	C_15_H_22_O_2_	235.16	Sesquiterpenoid	Anti-inflammatory agents; Tanaka et al. (2008)
16.43	342.14	Deoxymiroestrol	C_20_H_22_O_5_	343.15	Phenolic coumarin	Estrogenic activity; Udomsuk et al. (2011)
16.97	278.15	Emmotin A	C_16_H_22_O_4_	279.15	Sesquiterpenoid	Neurodegenerative diseases; Ngu et al. (2022)
17.47	229.20	2S-amino-tridecanoic acid	C_13_H_27_NO_2_	252.19	Long-chain fatty acid	Antimicrobial; Chowdhury et al. (2021)
18.04	286.21	2,3-Dihydroxycyclopentaneundecanoic acid	C_16_H_30_O_4_	304.24	Long-chain fatty acid	Antioxidant; Palakkal et al. (2017)
19.51	330.27	1-Monopalmitin	C_19_H_38_O_4_	353.26	Monoacylglycerole	
19.69	227.22	Halaminol A	C_14_H_29_NO	228.23	Amino alcohol	Anthelmintic activity; Herath et al. (2019)
20.20	208.14	(5alpha,8beta,9beta)-5,9-Epoxy-3,6-megastigmadien-8-ol	C_13_H_20_O_2_		Benzopyran	Xenobiotics; Dave et al. (2020)
20.42	224.21	3,7,11-Trimethyl-6E,10-dodecadien-1-ol	C_15_H_28_O	242.24	Terpene alcohol	
20.45	210.16	10-Tridecynoic acid	C_13_H_22_O_2_	228.19	Fatty acid	Antimicrobial; Krishnaveni et al. (2015)
20.81	255.25	Palmitic amide	C_16_H_33_N O	256.26	Amide	Anti-colon cancer cells; Wangchuk et al. (2014)
21.31	112.12	Cis-1,2-dimethylcyclohexane	C_8_H_16_	113.13	Cycloalkane	
22.19	283.28	Stearamide	C_18_H_37_N O	284.29	Amide	Lubricant; Briscoe et al. (1972)
23.24	448.49	8-Methyl-3-hentriacontene	C_32_H_64_	466.53	Aliphatic hydrocarbon	
24.11	426.37	Hexacosanedioic acid	C_26_H_50_O_4_	427.37	Dicarboxylic acid	
24.46	337.33	N-cyclohexanecarbonylpentadecylamine	C_22_H_43_NO	338.34	Amide	Acid amidase inhibitor; Tsuboi et al. (2004)

**RT**, retention time;**M. Mass**, molecular mass; **M. Formula**, molecular formula; **B. Peak**, base peak.

Several compounds were revealed in different classes, e.g., pyrimidine-dione, dimethyl xanthine, phenolic acids, carboxylic acid, hydroxycinnamic acid, stipitatonates, oxepanes, aldohexoses, pyranone, hydroxy acid, itaconic acid, disaccharide, galactolipids, inositol glycon, fatty acid, pyrrolidizine alkaloid, coumarin, phenoxyaceticacid derivative, sesquiterpenoid, monoacylglycerole, benzopyran, and dicarboxylic acid. Previous studies on various parts and extracts also indicated the presence of several compounds belonging to the classes of terpenoids, fatty acids, esters, and steroids by GCMS analysis; moreover, the presence of polyphenols was also observed by spectroscopic methods (Dilshad et al., 2022; Khalid et al., 2022). The occurrence of phenolic compounds was further established in another study by Akhter et al. (2021). This evidence signifies our results, showing the existence of various bioactive phytochemicals from fatty acids, esters, steroids, alcohols, and polyphenolic compounds.

These classes of chemical compounds showed a lot of biological and medicinal activities, e.g., mildly diuretic, respiratory stimulant, mutagenic agents, anti-inflammatory, analgesic, antioxidant, anticancer activity, vitamin D3, inhibition of hepatic cholesterol synthesis, antitumor, antimicrobial, gout, leukemia, anti-obesity, type 2 diabetes, estrogenic activity, P-GP-inhibitor, anthelmintic activity, anti-colon cancer cells, lubricant, and acid amidase inhibitor which are described in Table 1 and Table 2.

### 3.2 Polyphenolics profile by HPLC (quantitative analysis)

We evaluated the material toward 14 regularly used reference standards during our investigation. As a consequence, three major compounds were quantified in the sample: *p*-coumaric acid, 207.47 μg/g; gallic acid, 96.25 μg/g; and kaempferol, 95.78 μg/g (Table 3). A previous study on the roots and leaves of *T. domingensis* was found compatible with our results in which the presence of *p*-coumarates and ferulates was reported (He et al., 2015). It was further substantiated by a study previously performed on an aqueous extract of flowers from this plant showing the presence of phenols, hydroxycinnamic acids, flavonoids, and pro-anthocyanidins (Chai et al., 2014).

**TABLE 3:** HPLC quantification of TDME.

Component name	Peak #	RT (min)	Area	K-factor	Conc. (µg/g)
*p*-Coumaric acid	3	3.16	691,590.0	0.0003	207.47
Gallic acid	4	3.39	1093814.8	0.00088	96.25
Kaempherol	14	10.78	2,347,633.2	0.0000408	95.78

Natural substances called polyphenols are widely present in the foods and plants we frequently eat (Shams ul Hassan et al., 2022). They are essential for several biological processes that take place in the seeds, leaves, roots, and other plant tissues. These include controlling enzyme activity, regulating protein synthesis, promoting photosynthesis, and maintaining the cytoskeleton’s structural integrity (Zhang et al., 2019). The powerful analytical method known as HPLC is utilized to identify and measure the chemical components in samples. It enables the exact and accurate measurement and identification of various chemicals. HPLC can also be used to separate and gather particular quantities of individual compounds by placing a fraction collector behind the detector’s flow cell, allowing their purification and collection (Javed et al., 2020).

Among the discovered polyphenols from TDME, *p*-coumaric acid belongs to the hydroxycinnamic acid group and is classified as a phenolic acid with anti-inflammatory, antidiabetic, antioxidant, anti-platelet, anti-ulcer, and anti-cancer characteristics (Ilavenil et al., 2016). Gallic acid is frequently found in antioxidant tea formulations and Ayurvedic plants. It has several health advantages, including antioxidant qualities, anti-inflammatory properties, and possibly anti-cancer potential (Aqeel et al., 2023). Gallic acid, a trihydroxy benzoic acid found in plant metabolites all over the world, is effective in the treatment of gastrointestinal, cognitive, metabolic, and cardiovascular problems. It can defend biological cells, tissues, and organs from damage brought on by oxidative stress because of its outstanding antioxidant and free radical scavenging activities (Kahkeshani et al., 2019).

A polyphenol antioxidant called kaempferol belongs to the flavonoids class and is found in medicinal plants, some fruits, and vegetables. Studies have repeatedly shown that eating foods high in kaempferol can lower the risk of getting chronic illnesses. The body’s antioxidant defenses are strengthened by kaempferol because it fights free radicals. Additionally, it influences crucial processes like metastasis, angiogenesis, inflammation, and apoptosis (Chen and Chen, 2013).

### 3.3 *In vitro* biological investigation

#### 3.3.1 Enzyme inhibition activities (lipoxygenase, α-glucosidase, and urease)

The results of the current study showed good results of enzyme inhibitions (Table 4). The lipoxygenase (44.75 ± 0.51 µgmL^-1^), α-glucosidase (52.71 ± 0.01 µgmL^-1^), and urease (67.19 ± 0.68 µgmL^-1^) inhibition showed significant results of TDME, which were comparable to the standards used (indomethacin, 18.03 ± 0.12 µgmL^-1^; quercetin, 4.11 ± 0.01 µgmL^-1^; thiourea, 8.97 ± 0.11 µgmL^-1^). Our results were also in agreement with evidence by Sen et al. on lipoxygenase inhibition by the methanolic extract of another *Typha* species (*Typha elephantina*). Lipoxygenases (LOX) are enzymes that play an important part in the manufacture of leukotrienes, which are bioactive lipids. Their strength resides in their involvement in several physiological processes such as inflammation (Abbas et al., 2022), immune response control, and cell signaling, making them promising therapeutic targets in disorders such as asthma and other inflammatory ailments. Furthermore, the ability of lipoxygenases to catalyze lipid oxidation events increases their importance in lipid metabolism and overall cellular homeostasis (Sajid-ur-Rehman et al., 2023).

**TABLE 4:** Enzyme nhibition activities of TDME.

Extract	Lipoxygenase IC_50_ (µgmL^-1^) ± SD	α-Glucosidase IC_50_ (µgmL^-1^) ± SD	Urease IC_50_ (µgmL^-1^) ± SD
**TDME**	44.75 ± 0.51	52.71 ± 0.01	67.19 ± 0.68
**Standard**	18.03 ± 0.12^a^	4.11 ± 0.01 ^b^	8.97 ± 0.11^c^

Values are expressed as the mean ± SD of three parallel measurements. “a” represents indomethacin, “b” represents quercetin, and “c” represents thiourea.

According to the previous study, *T. domingensis* is an excellent source of glucosidase inhibitors, iron chelators, and natural antioxidants. Oxidative stress is also a central consideration during the management of diabetes, and many bioactive phytochemicals including polyphenols are acknowledged as potent antioxidants (Ahmed et al., 2022b). The plant’s fruit extract is more effective as an anti-glucosidase and antioxidant than the male and female flower extract. In contrast, the female floral extract has the greatest ability to chelate iron among the three extracts. The phenolic content of the extract can be credited with both antioxidant and anti-glucosidase properties. Our research offers a molecular explanation for TDME’s capacity to treat diabetes and promote wound healing (Chai et al., 2014). This antioxidant potential is primarily due to the inhibition of pro-inflammatory mediator release, free radical neutralization, ROS, and RNS, which reduces lipid peroxidation and activates the cyclooxygenase pathway (Khatri et al., 2019). Additionally, they influence blood glucose levels by several methods, including limiting intestinal glucose absorption, promoting cell insulin production, and improving glucose uptake in insulin-sensitive tissue (Vinayagam et al., 2016).

Anti-urease drugs are now focusing on their potent anti-ulcer properties. Urease enzyme activity has been identified as a key pathogenic determinant in the etiology of several serious disorders that are harmful to human health, animals, and agriculture [67]. Sesquiterpene hydrocarbons or alcohols, or their synergistic action, may have very strong antibacterial urease activity. The primary defense against *Helicobacter pylori* may be due to urease inhibition, which prevents the bacteria from adhering to the gastric mucosa. The process of urease inhibition was discovered to be non-competitive, with both the substrate and inhibitor being incompetently attached to the enzyme.

#### 3.3.2 Hemolytic activity to investigate the *in vitro* safety of TDME

Data on the hemolytic activity of the TDME are shown in Table 5. TDME has a hemolytic percentage value of 4.25 0.16%, and the cytotoxic positive control showed significantly high hemolysis (93.51% ± 0.36%). Previously, many methanolic extracts from plants were tested by the hemolytic activity to establish their possible safety, and our results fall in a similar range (Tabassum et al., 2023). The ability of some chemicals, such as toxins or enzymes, to cause the lysis or destruction of RBCs is referred to as hemolytic activity. This characteristic is useful in a variety of research and diagnostic applications, such as determining pathogen pathogenicity, researching the impact of certain substances on blood cells, and identifying specific medical problems associated with aberrant hemolysis. It can, however, be a negative component, creating health problems when detected in certain pathogenic agents or poisons. According to the reported methods, the hemolysis activity of less than 30% is considered safe and non-toxic for humans (Tabassum et al., 2022).

**TABLE 5:** Hemolytic activities to assess the safety and anti-inflammatory action of TDME.

Extract	Hemolysis (%)	Hemolysis inhibition (%)
TDME	4.25 ± 0.16	39.16 ± 1.33
Standards	93.51 ± 0.36^a^	55.91 ± 1.78^b^

For each experiment, the process was performed three times. The data are shown as the mean ± standard deviation (n = 3). ^a^Triton × 100 serves as the reference standard in hemolytic activity for safety analysis; ^b^diclofenac sodium serves as standard to investigate anti-inflammatory properties.

#### 3.3.3 *In vitro* anti-inflammatory activity

Water-induced lysis of RBCs was significantly inhibited by TDME (Table 5). The results of percentage hemolysis inhibition showed a value of 39.16% ± 1.33%, compared to the standard inhibitor of inflammation (diclofenac sodium) in this study, which showed an inhibition value of 55.91% ± 1.78% at the dose of 1,000 μg/mL. It was reported that the denaturation of protein cells and tissue injury are reflected in good correlation with inflammatory diseases [28]. This finding demonstrates the anti-inflammatory action of TDME. Several compounds identified in the current study (Table 1; Table 2; Table 3) may be responsible for this activity.

### 3.4 *In vivo* biological investigation

#### 3.4.1 Acute toxicity assay

The TDME was shown to be non-toxic up to a dosage of 10 g/kg in acute toxicity experiments on rates to assess the potential negative effects of increasing doses of TDME. No poisoning symptoms were noticed, and no deaths were reported over the 48-h observation period.

#### 3.4.2 Anti-inflammatory activity (carrageenan-induced paw edema)

**FIGURE 4:**
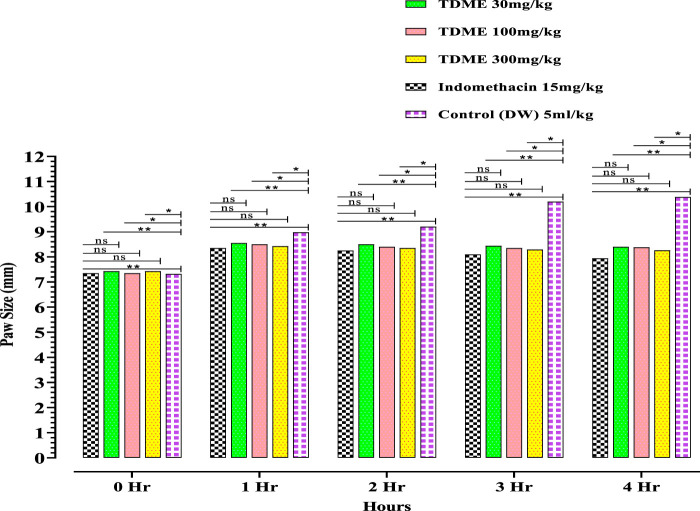
Carrageenan-induced paw edema of TDME.

All dose levels of TDME showed anti-inflammatory effects (Figure 4 and Table 6). Notably, after carrageenan administration, significant anti-inflammatory effects from TDME were observed during the third and fourth hours. The 300 mg/kg dose had the strongest effect during the fourth hour of the doses examined. Carrageenan is a potent inflammatory agent used to induce edema. Various inflammatory mediators are involved in the development of carrageenan paw edema, and that has been extensively used to assess the anti-edema effects of natural products [69]. In the current study, various phytochemicals, especially polyphenols (Table 1; Table 2; Table 3), may be contributing to the reduction of inflammation. Moreover, several phytochemicals identified by HPLC–MS also have reported anti-inflammatory effects (Table 1; Table 2), which also shows a probability of their participation in declining inflammation.

**TABLE 6:** Effect of TDME and indomethacin in carrageenan-induced paw edema test in rats.

Treatment	Paw size (mm)				
	0 hr	1 hr	2 hr	3 hr	4 hr
Control (distilled water (DW)) (5 mL/kg)	7.32 ± 0.16	8.98 ± 0.15	9.20 ± 0.20	10.20 ± 0.20	10.38 ± 0.18
Indomethacin (15 mg/kg)	7.35 ± 0.16	8.35 ± 0.15	8.25 ± 0.15***	8.10 ± 0.10***	7.94 ± 0.13***
TDME (30 mg/kg)	7.43 ± 0.13	8.55 ± 0.21	8.50 ± 0.21 **	8.44 ± 0.20***	8.40 ± 0.19***
TDME (100 mg/kg)	7.35 ± 0.18	8.50 ± 0.20	8.40 ± 0.21 **	8.35 ± 0.21***	8.30 ± 0.25***
TDME (300 mg/kg)	7.43 ± 0.15	8.43 ± 0.18	8.35 ± 0.16 **	8.29 ± 0.19***	8.26 ± 0.18***

All values are presented as mean ± SD. *p* < 0.001 (***) and *p* < 0.05 (**) *versus* the control. A two-way ANOVA was used in the statistical analysis, followed by Bonferroni’s test.

#### 3.4.3 Analgesic activity

##### 3.4.3.1 Hot plate test

**FIGURE 5:**
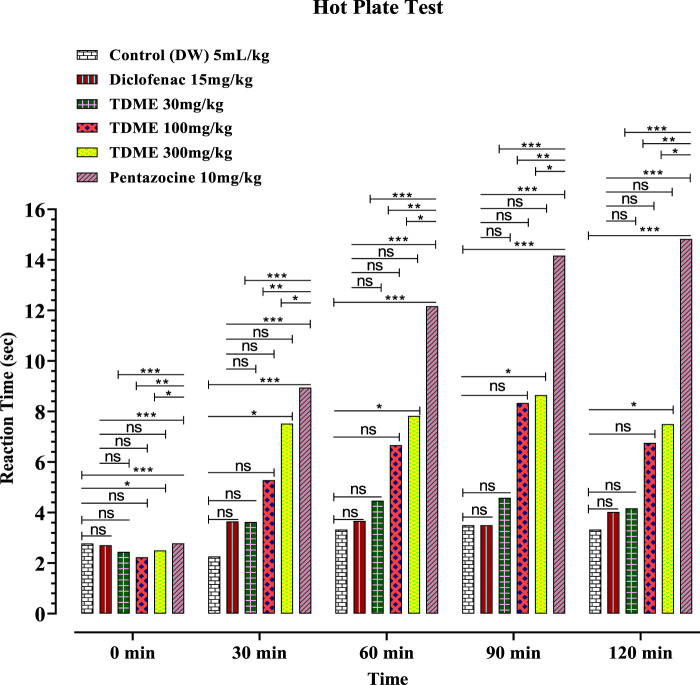
Hot plate test of TDME.

The hot plate test, like the tail-flick test, assesses the pain response in animals. Both the hot plate and tail immersion methods are commonly used to assess centrally acting analgesics [70]. Pentazocine and diclofenac sodium, standard drugs, substantially reduced pain latency at 30, 60, 90, and 120 min, whereas TDME at 30 mg/kg had no significant effect on pain reduction at any of the observation intervals (Figure 5 and Table 7). When compared to the control group, TDME at 100 and 300 mg/kg doses significantly reduced pain latency at all monitoring intervals (30, 60, 90, and 120 min) as compared to the control group.

**TABLE 7:** Effects of diclofenac, pentazocine, and TDME on the hot plate test in rats.

Treatment	Reaction time (sec)				
	0 min	30 min	60 min	90 min	120 min
Control (DW) (5 mL/kg)	2.78 ± 0.31	2.27 ± 0.21	3.33 ± 0.33	3.50 ± 0.22	3.33 ± 0.42
Diclofenac (15 mg/kg)	2.71 ± 0.21	3.65 ± 0.36	3.67 ± 0.21	3.50 ± 0.34	4.03 ± 0.25
Pentazocine (10 mg/kg)	2.79 ± 0.3	8.95 ± 0.43***	12.17 ± 0.65***	14.17 ± 0.65***	14.83 ± 0.70***
TDME (30 mg/kg)	2.45 ± 0.22	3.63 ± 0.49***	4.47 ± 0.73***	4.59 ± 0.55***	4.17 ± 0.40***
TDME (100 mg/kg)	2.24 ± 0.21	5.29 ± 1.02***	6.67 ± 0.94***	8.34 ± 0.95***	6.76 ± 0.75***
TDME (300 mg/kg)	2.50 ± 0.21	7.52 ± 0.65***	7.83 ± 0.87***	8.65 ± 0.76***	7.50 ± 0.88***

The longer latency time observed in the hot plate test on Wistar albino rats implies that various doses of TDME, diclofenac, and pentazocine have analgesic effects compared to –ve control distilled water. All values are given in mean ± SEM. N = 6. *p* < 0.001 (***), as compared to control (two-way ANOVA followed by Bonferroni’s test).

##### 3.4.3.2 Tail immersion test

**FIGURE 6:**
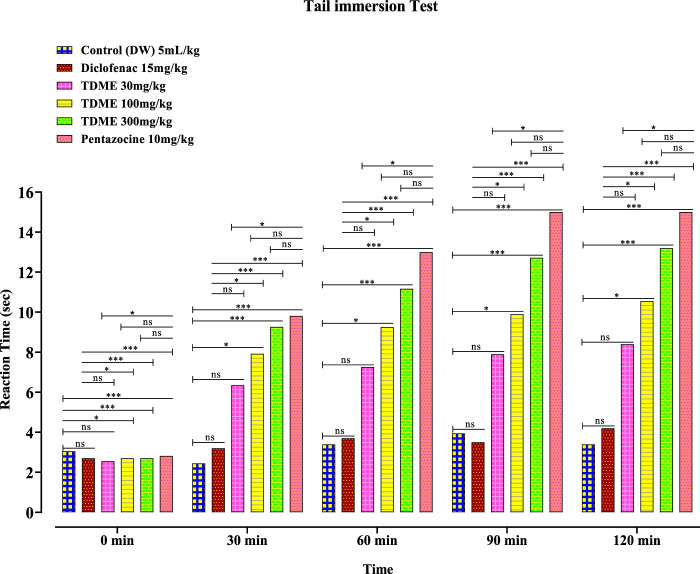
Tail immersion test of TDME.

An additional measure used to assess a substance’s analgesic potential is the tail immersion test. All three doses of TDME (30, 100, and 300 mg/kg) significantly decreased pain latency when compared to the control group (Figure 6 and Table 8). The tail immersion assay is a thermally based test used to assess a substance’s analgesic capabilities. This particular test has the advantage of not being sedation-affected, unlike other assays like the hot plate test, and is known for evaluating pain-related responses that are predominantly driven by spinal mechanisms. A unique sensitivity to partial agonists and narcotic agonists was seen in the tail immersion test performed on rats. In light of this, it is advised to use this test to assess the antinociceptive efficacy of partial agonists in small laboratory animals (Javed et al., 2020).

**TABLE 8:** Tail immersion test of TDME.

	Reaction time (sec)				
Treatment	0 min	30 min	60 min	90 min	120 min
Control (distilled water (DW)) (5 mL/kg)	3.05 ± 0.24	2.45 ± 0.23	3.40 ± 0.35	3.59 ± 0.22	3.40 ± 0.42
Diclofenac (15 mg/kg)	2.70 ± 0.20	3.20 ± 0.33	3.70 ± 0.25	3.50 ± 0.32	4.2 ± 0.22
Pentazocine (10 mg/kg)	2.82 ± 0.35	9.82 ± 0.49***	13 ± 0.68***	15 ± 0.63***	15 ± 0.70***
TDME (30 mg/kg)	2.55 ± 0.25	6.35 ± 0.53***	7.26 ± 0.42***	7.9 ± 0.63***	8.40 ± 0.45***
TDME (100 mg/kg)	2.70 ± 0.21	7.92 ± 0.72***	9.25 ± 0.63***	9.90 ± 0.85***	10.55 ± 0.62***
TDME (300 mg/kg)	2.70 ± 0.23	9.27 ± 0.92***	11.17 ± 0.72***	12.72 ± 0.45***	13.2 ± 0.31***

All values are given in mean ± SEM. N = 6. *p* < 0.001 (***) as compared to the control (two-way ANOVA followed by Bonferroni’s test).

### 3.5 *In silico* prediction studies

#### 3.5.1 Molecular docking

The crystal structures of α-glucosidase (PDB: 3WY1), urease (PDB: 1EJX), 5-lipoxygenase LOX (PDB: 6NCF), and cyclooxygenases (COX1, PDB: 6y3c and COX2, PDB: 1CX2) were obtained from https://www.rcsb.org/. The ligands were chosen from RP-UHPLC–MS, and their structures were downloaded from PubChem.

**FIGURE 7:**
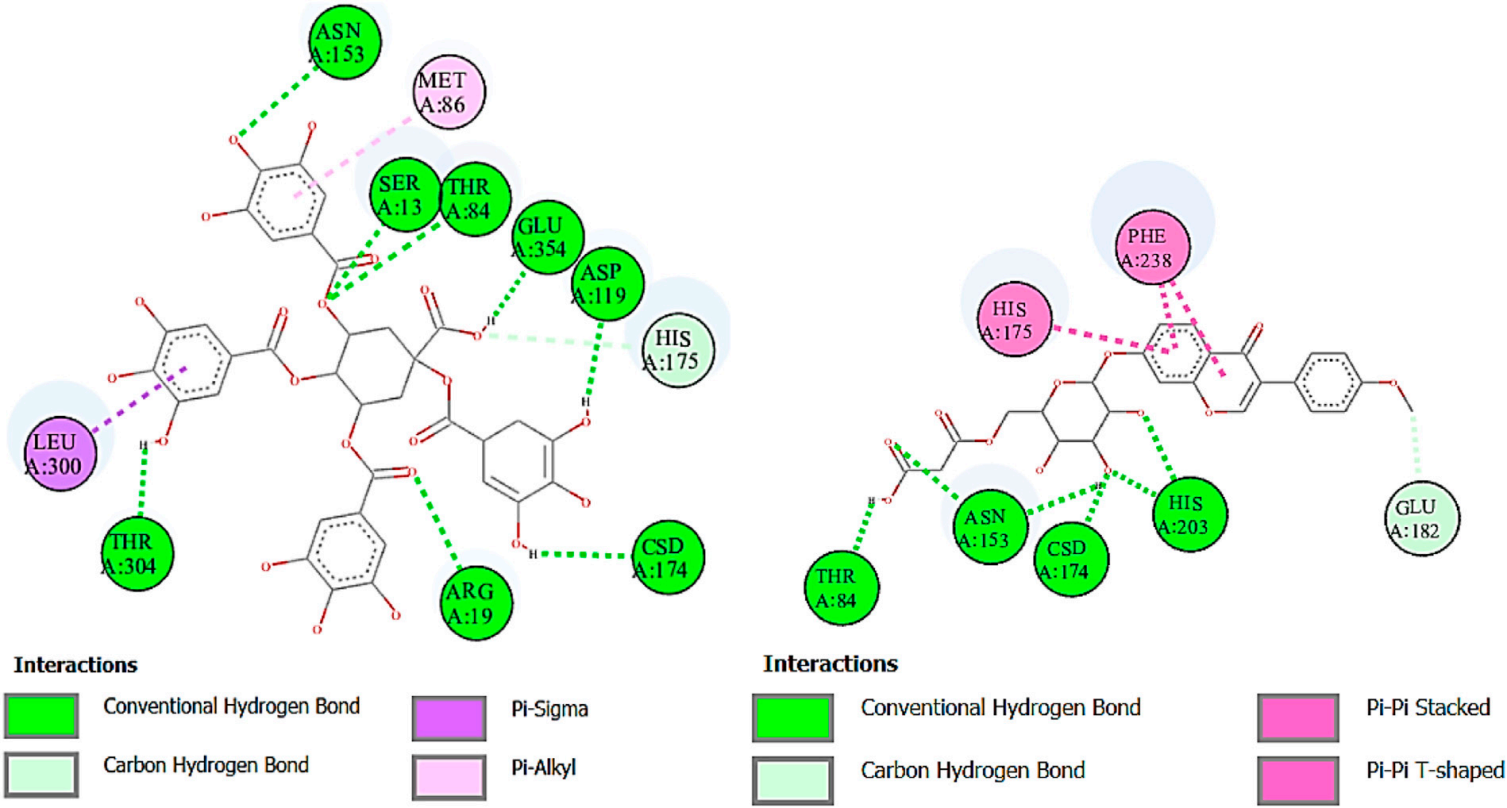
Two-dimensional interactions of 1,3,4,5-tetracaffeoylquinic acid (left) and formononetin 7-O-glucoside-6″-O-malonate (right) against α-glucosidase.

α-Glucosidase inhibitors are used for the treatment of type 2 diabetes to slow down the digestion and absorption of carbohydrates, helping to regulate blood sugar levels (Kashtoh and Baek, 2022). The ligands from RP-UHPLC–MS showed very good binding affinity against α-glucosidase. 1,3,4,5-Tetracaffeoylquinic acid (−9.2), deoxymiroestrol (−8.1), formononetin 7-O-glucoside-6″-O-malonate (−8.0), stipitatonate (−6.9), N-cyclohexanecarbonylpentadecylamine (−6.9), 4-methylumbelliferone (−6.8), nigerose (sakebiose) (−6.7), vanillic acid 4-sulfate (−6.5), emmotin A (−6.6), vanillic acid 4-sulfate (−6.5), nonic acid (−5.6), annuionone B (−5.4), L-galactose (−5.9), purine (−5.4), curcumenol (−6.4), theobromine (−5.7), ethyl 4-methylphenoxyacetate (−6.3), 3-eydroxy-3-methyl-glutaric acid (−5.7), and curcumenol (−6.4) are presented in Table 9. The higher negative values of the binding energy score represent the higher binding affinity of the ligand with the receptor molecule, which may be due to the chemical structures and interactions of compounds with amino acid residues (Ahmed et al., 2022a). The 2D interactions of α-glucosidase with ligands having the highest docking score are shown in Figure 7. The standard used for α-glucosidase was quercetin. The previous study also showed that the fruit and female flower extracts of *T. domingensis* are promising sources of glucosidase inhibitors (Chai et al., 2014).

**TABLE 9:** Interaction of different ligands with α-glucosidase, urease, and cyclooxygenase enzyme.

Compound name	α-Glucosidase		Urease		Lipoxygenase (LOX)		Cyclooxygenase COX-1		Cyclooxygenase COX-2	
	DFR in Kcal/mol	Interaction	DFR in Kcal/mol	Interaction	DFR in Kcal/mol	Interaction	DFR in Kcal/mol	Interaction	DFR in Kcal/mol	Interaction
Formononetin 7-O-glucoside-6″-O-malonate	−8	**HB:** Leu227 and Asn301	−8.7	**HB:** Pro1300, Thr1305, Leu1558	−7.9	**HB:** Phe555, Ser608, Tyr660, Arg666	−9.4	**HB:** Thr206, Tyr385, Tpr387, His388	−10.3	**HB:** Asn39, Arg44, Glu46, Cys47, Pro154, Gln461
		**π π:** Ala224								**π π:** Leu152, Lys468, Arg469
1.3,4,5-Tetracaffeoylquinic acid	−9.2	**HB:** Thr226, Ala229, Glu231, Asn301, Glu377, Ala378, and Gly399	−7.7	**HB:** Ser1436, Lys1443, Pro1444, Ala1445, Arg1563, Tyr1564	−12.0		−10.7	**HB:** Tyr148, Thr212, Met379	−13.1	
		**π π:** Pro230, and Val334		**π π:** Pro1457				**π π:** Ala202, Lys211, Val291, Val451		
Quercetin (standard)	**−8**	**HB:** Leu227, Leu227, Met302, Glu396								
		**π π:** Pro230, and Val334								
Hydroxyurea (standard)			−4.9	**HB:** Gly1277, Glu1274, Thr1298, Glu1345						
Indomethacin (standard)					−7.4	**HB:** Val112	−7.4	**HB:** Asn382, Tyr385	−8.0	**HB:** Tyr122, Ser471
										**π π:** Arg44, Arg469, Pro474

**HB**, hydrogen bonding; **π** and **π**, pi alkyl bond.

**FIGURE 8:**
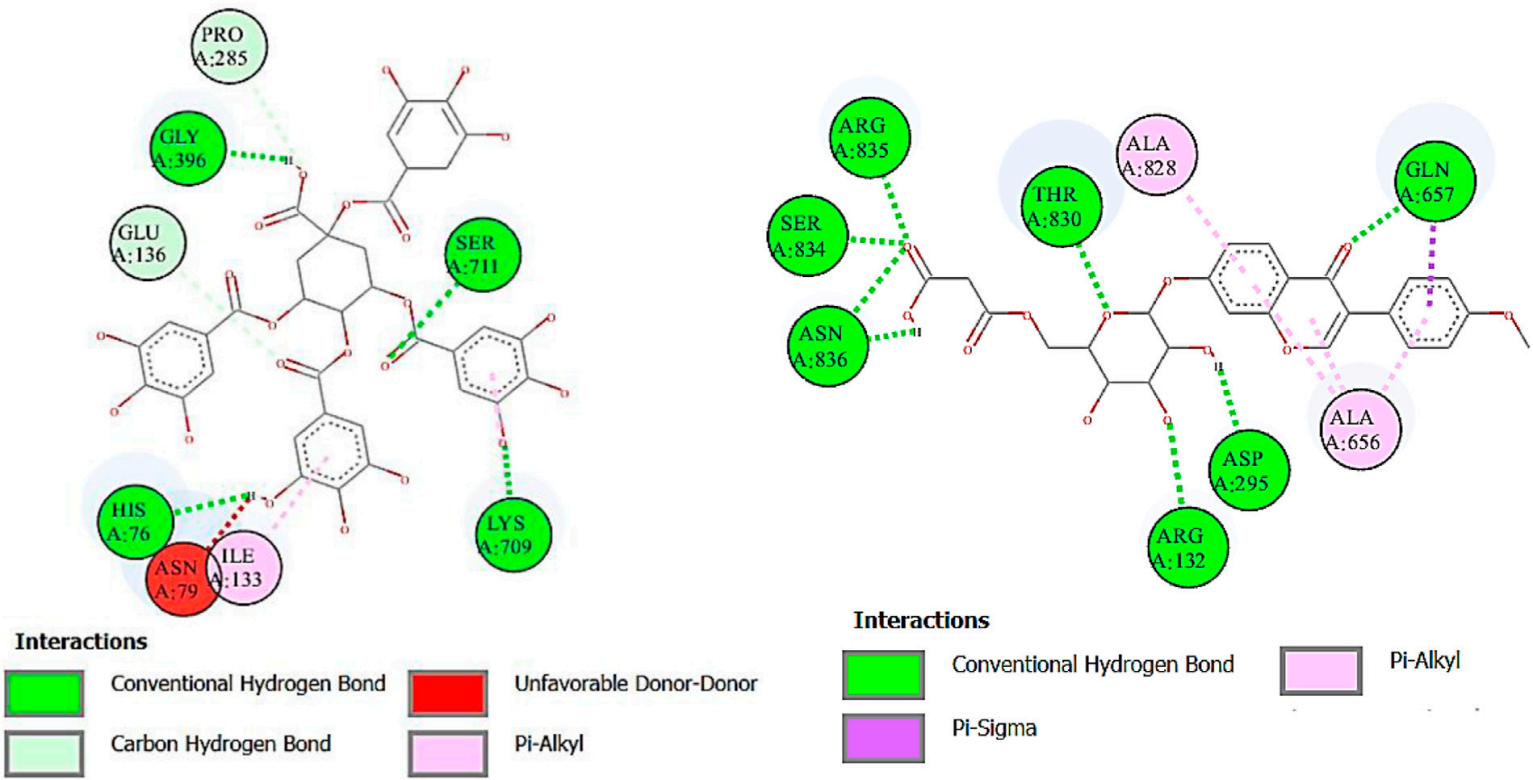
Two-dimensional interactions of 1,3,4,5-tetracaffeoylquinic acid (left) and formononetin 7-O-glucoside-6″-O-malonate (right) against urease.

Urease is used to determine the *H. pylori* infection, a common cause of gastritis and gastrointestinal ulcers. In-depth research on diverse classes of urease inhibitors has been provoked by the need to treat such illnesses (Kosikowska and Berlicki, 2011). A lot of ligands showed the strongest binding affinity like formononetin 7-O-glucoside-6″-O-malonate (8.7), deoxymiroestrol (−8.1), 1.3,4,5-tetracaffeoylquinic acid (−7.7), stipitatonate (−6.7), 6-(alpha-D-glucosaminyl)-1D-myo-inositol (−6.7), vanillic acid 4-sulfate (−6.6), codonopsine (6.5), l5-acetylamino-6-formylamino-3-methylurssacil (−6.4), emmotin A (−6.4), nonic acid (−4.9), annuionone B (−6.0), L-galactose (−5.5), purine (−5.1), gurcumenol (−6.7), theobromine (−5.4), ethyl 4-methylphenoxyacetate (−5.8), 3-eydroxy-3-methyl-glutaric acid (−5.0), and nigerose (6.3), as presented in Table 9. The 2D interactions of ligands with the highest docking score against urease are presented in Figure 8.

**FIGURE 9:**
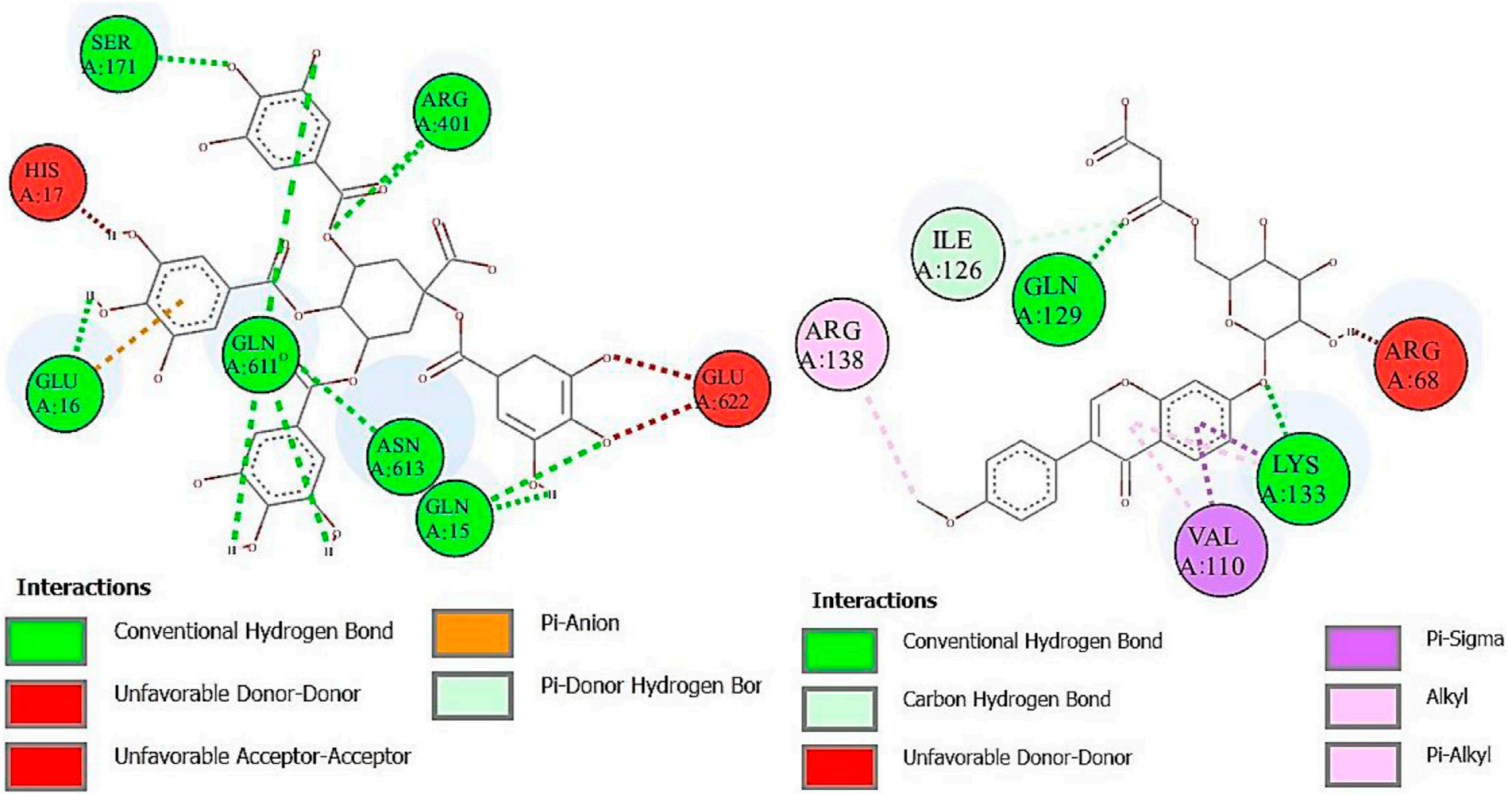
Two-dimensional interactions of 1,3,4,5-tetracaffeoylquinic acid (left) and formononetin 7-O-glucoside-6″-O-malonate (right) against lipoxygenase (5-LOX).

Lipoxygenase (LOX) is an oxidoreductase enzyme that is found in both plants and animals. Its enzymatic action results in the formation of aromatic molecules, making it useful in the production of pleasant fragrances. Furthermore, LOX regulates the synthesis of volatile compounds, making it a great natural taste enhancer in the food preparation process (Steele et al., 2000). The binding affinity of deoxymiroestrol (−8.6), N-cyclohexanecarbonylpentadecylamine (−7.3), emmotin A (−7.0), and vanillic acid 4-sulfate (−7.4) showed good results. Indomethacin standard was used for LOX. Formononetin 7-O-glucoside-6″-O-malonate and 1,3,4,5-tetracaffeoylquinic acid showed better binding affinity as compared to the standard compound. The 2D interactions of ligands with the highest docking score against lipoxygenase are presented in Figure 9.

**FIGURE 10:**
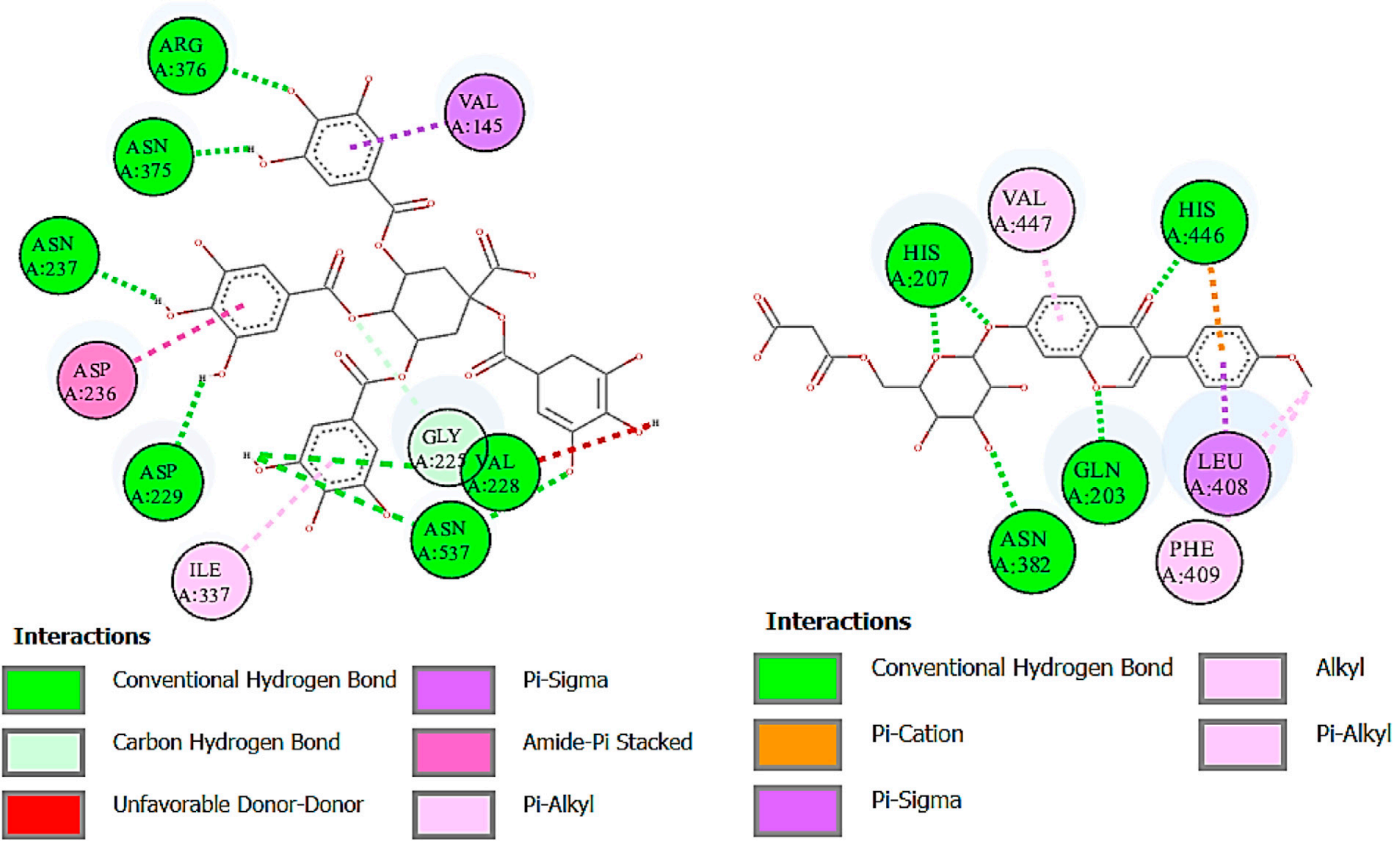
Two-dimensional interactions of 1,3,4,5-tetracaffeoylquinic acid (left) and formononetin 7-O-glucoside-6″-O-malonate (right) against cyclooxygenase-1 (COX-1).

**FIGURE 11:**
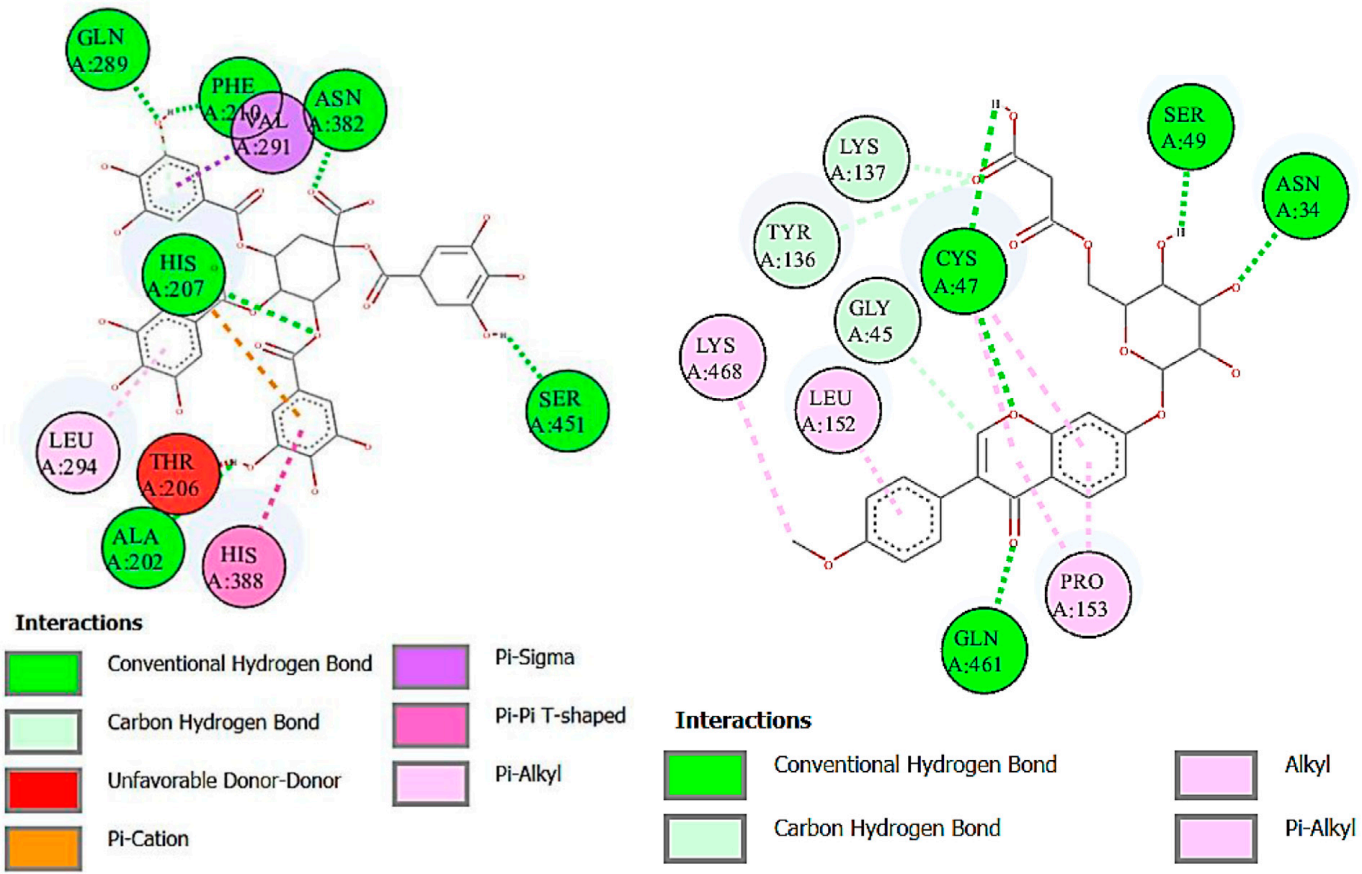
Two-dimensional interactions of 1,3,4,5-tetracaffeoylquinic acid (left) and formononetin 7-O-glucoside-6″-O-malonate (right) against cyclooxygenase-2 (COX-2).

Cyclooxygenase COX-1 inhibitors are used for the reduction of inflammation and also anti-platelet properties. Cyclooxygenase COX-2 inhibitors are used to alleviate pain and inflammation while minimizing gastrointestinal side effects compared to traditional nonsteroidal anti-inflammatory drugs (NSAIDs) (Ahmad et al., 2018). The binding affinity of deoxymiroestrol (COX-1, -8.0 and COX-2, -8.7), N-cyclohexanecarbonylpentadecylamine (COX-1, -7.0 and COX-2, -8.3), emmotin A (COX-1, -7.4 and COX-2, -7.3), and vanillic acid 4-sulfate (COX-1, -6.5 and COX-2, -6.8) is shown. The standard used for cyclooxygenase was indomethacin. Many compounds showed results higher than standard. The 2D interactions of ligands with the highest docking score against COX-1 and COX-2 are presented in Figure 10 and Figure 11, respectively.

#### 3.5.2 *In silico* absorption, distribution, metabolism, excretion, and toxicological characterization of compounds

All dots showed within pink color which is near to target. So, this is called an optimized drug. Smaller TPSA values (usually below 140–160 Å^2^) are frequently chosen for oral bioavailability in drug development, for instance, as bigger TPSA values may result in decreased permeability through cell membranes. On the other hand, compounds designed to engage with particular targets or receptors may benefit from having higher TPSA values (over 80–100 Å^2^). Typically, the molar refractivity range is 0–100 cm³/mol. The range of consensus Log P o/w is between −3 and +6. The solubility range is < −10 < poorly < −6 < moderately < −4 < soluble < -2 very <0 < highly. Skin permeation range is −1.2 to +1.32. If any compound follows at least three rules of drug-likeness, it can act as an oral drug (Shahid et al., 2023). To determine pharmacokinetic drug features, such as absorption, distribution, metabolism, and excretion, the Lipinski rule makes use of certain physicochemical qualities. The product’s molecular weight should not exceed 500 Da. The number of atoms in its molecules should range between 20 and 70, with an average of 50. The polar surface area must be less than 140 Å^2^. Furthermore, the product should not contain more than five hydrogen bond donor sites and not surpass 10 hydrogen bond acceptor sites (Lipinski et al., 2012). The results of ADME and toxicological studies are presented in Table 10 and 11, respectively.

**TABLE 10:** Pharmacokinetics, bioavailability, structural parameters, Lipinski rule violation count, and solubility predictions of major compounds from TDME.

Compound name and their bioavailability radar	Physicochemical property	Lipophilicity	Water solubility	Pharmacokinetics	Drug-likeness		
Curcumenol 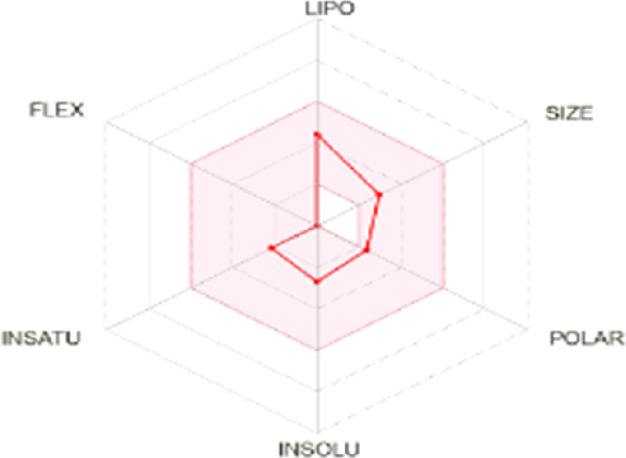	MR: 69.25; TPSA: 29.46 A^2^	C log P 0/w:2.91	Log S (E Sol): -2.70; Log S (Ali): -2.49; Log S (SILICOS-IT): -2.67	GI Abs: high; BBB: yes; P-gp substrate: no; CYP1A2 inhibitor; Log Kp: -6.14 cm/s	Lipinski 0 violation: yes	HBD: 1; HBA: 2	
					Ghose: yes		
					Veber: yes		
					Egan: yes		
					Muegge: yes		
					B score: 0.55		
Deoxymiroestrol 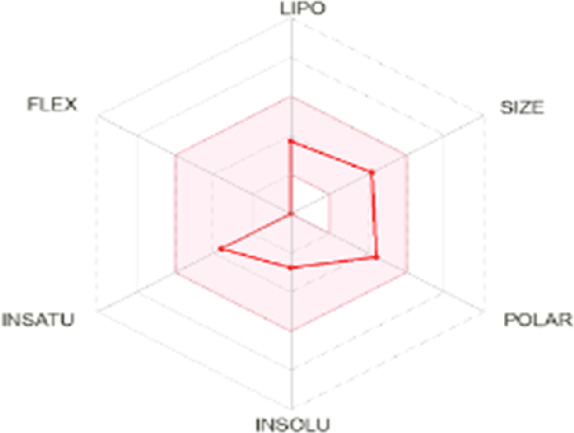	MR: 90.98; TPSA: 86.99 A^2^	C log P 0/w: 1.66	Log S (E Sol): -2.75; Log S (Ali): -2.37; Log S (SILICOS-IT): -2.94	GI Abs: high; BBB: no; P-gp substrate: yes; CYP1A2 inhibitor: no; Log Kp: -7.71 cm/s	Lipinski 0 violation: yes	HBD: 3; HBA: 5	
					Ghose: yes		
					Veber: yes		
					Egan: yes		
					Muegge: yes		
					B score: 0.55		
Ethyl 4-methylphenoxyacetate 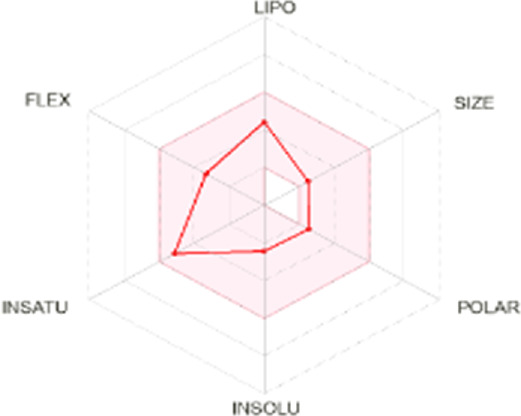	MR: 53.61; TPSA: 35.53 A^2^	C log P 0/w: 2.13	Log S (E Sol): -2.45; Log S (Ali): -2.63; Log S (SILICOS-IT): -3.41	GI Abs: high; BBB: yes; P-gp substrate: no; CYP1A2 inhibitor: yes; Log Kp: -5.89 cm/s	Lipinski 0 violation: yes	HBD: 0; HBA: 3	
					Ghose: yes		
					Veber: yes		
					Egan: yes		
					Muegge: no. 1 violation MW < 200		
					B score: 0.55		
Theobromine 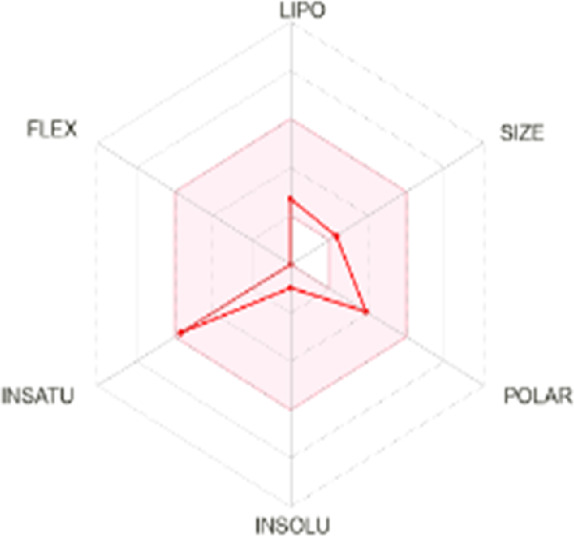	MR: 47.14; TPSA: 72.68 A^2^	C log P 0/w: -0.20	Log S (E Sol): -0.98; Log S (Ali): -0.27; Log S (SILICOS-IT): -1.10	GI Abs: high; BBB: no; P-gp substrate: no; CYP1A2 inhibitor: no; Log Kp: -7.95 cm/s	Lipinski 0 violation: yes	HBD: 1; HBA: 3	
					Ghose: no. 1 violation WLOPG < −0.4		
					Veber: yes		
					Egan: yes		
					Muegge: no. 1 violation MW < 200		
					B score: 0.55		
3-Hydroxy-3-methyl-glutaric acid 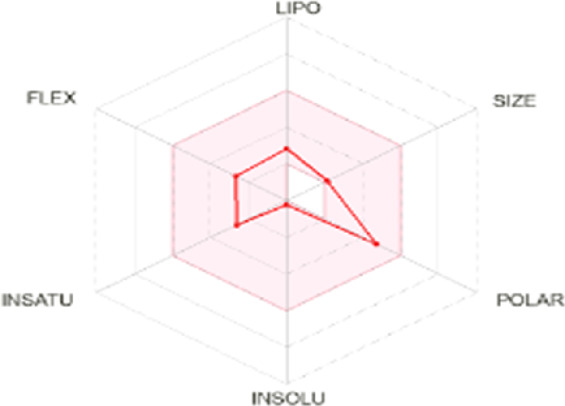	MR: 35.66; TPSA: 94.83 A^2^	C log P 0/w: -0.40	Log S (E Sol): -0.24; Log S (Ali): -0.98; Log S (SILICOS-IT): 1.07	GI Abs: high; BBB: no; P-gp substrate: no; CYP1A2 inhibitor: no; Log Kp: -7.67 cm/s	Lipinski 0 violation: yes	HBD: 3; HBA: 5	
					Ghose: 2 violation: WLOGP < -0.4, MR < 40		
					Veber: yes		
					Egan: yes		
					Muegge: no. 1 violation: MW < 200		
					B score: 0.56		
Nonic acid 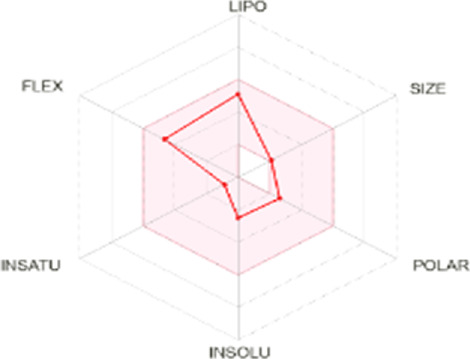	MR: 47.15; TPSA: 37.30 A^2^	C log P 0/w: 2.60	Log S (E Sol): -2.51; Log S (Ali): -3.88; Log S (SILICOS-IT): -2.46	GI Abs: high; BBB: yes; P-gp substrate: no; CYP1A2 inhibitor: no; Log Kp: -4.84 cm/s	Lipinski 0 violation: yes	HBD: 2; HBA: 4	
					Ghose: no. 1 violation: MW < 200		
					Veber: yes		
					Egan: yes		
					Muegge: no. 1 violation: MW < 200		
					B score: 0.85		
Annuionone B 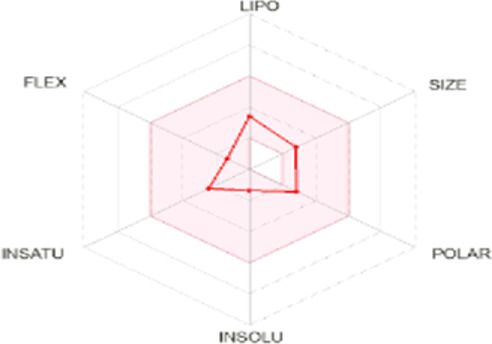	MR: 61.17; TPSA: 43.37 A^2^	C log P 0/w: 1.72	Log S (E Sol): -1.36; Log S (Ali): -0.92; Log S (SILICOS-IT): -2.40	GI Abs: high; BBB: yes; P-gp substrate: no; CYP1A2 inhibitor: no; Log Kp: -7.34 cm/s	Lipinski 0 violation: yes		HBD: 0; HBA: 3
					Ghose: yes		
					Veber: yes		
					Egan: yes		
					Muegge: yes		
					B score: 0.55		

**MR**, molar refractivity; **TPSA**, topological polar surface area, **C log**, consensus log P; **Log S (E Sol):** estimated solubility; **S by Ali et al.**, solubility by Ali et al.; **SF by IT P**, solubility filter by IT, programmed; **GI Abs**, gastrointestinal track; **P-gp substrate**, P glycoprotein substrate, **BBB permeant**, blood–brain barrier; **CYP1A2 inhibitor**, cytochrome P450 1A2 inhibitor; **Log Kp**, skin permeation; **B score**, bioavailability score; **HBA**, hydrogen bond acceptor; **HBD**, hydrogen bond donor.

## 4 Conclusion

The study represents the chemical constitution and biological activities (*in vitro, in vivo*, and *in silico*) of the methanolic extract of *T. domingensis* (TDME). The result of RP-UHPLC–QTOF-MS screening revealed the presence of various classes of bioactive compounds. HPLC quantification offered a reliable and adaptable approach, enabling precise analysis and facilitating a diversity of academic and industrial applications. The inhibition of lipoxygenase, urease, and α-glucosidase offer considerable potential in the development of innovative treatment approaches for gastrointestinal diseases and metabolic disorders. The detected hemolytic activity of the TDME has disruptive effects on RBCs and suggests its importance in both research and therapeutic settings to validate the safety of the analyte, which was further confirmed by the *in vivo* acute toxicity method. Both *in vitro* and *in vivo* experiments showed significant analgesic and anti-inflammatory effects of TDME. Hence, this recent research provides support for the plant’s efficacy in alleviating pain and addressing inflammatory diseases. This research highlights the therapeutic potential of TDME, which could be further investigated for its applications in the nutraceutical and pharmaceutical industries.

## Data Availability

The original contributions presented in the study are included in the article/Supplementary Material; further inquiries can be directed to the corresponding authors.
