# Supplementary material for: Phytochemical characterization of Typha domingensis and the assessment of therapeutic potential using in vitro and in vivo biological activities and in silico studies
Source: Front Chem. 2023 Nov 8;11:1273191. doi: 10.3389/fchem.2023.1273191 (PMC10663946; doi:10.3389/fchem.2023.1273191)
Supplement: Supplementary file 1 [file DataSheet1.pdf]

Supplementary Material

Phytochemical Characterization of Typha domingensis Extract by HPLC and Assessment of its Therapeutic Potential through In-vitro Biological, In-vivo Anti-inflammatory and Analgesic Activities, and In-silico Study of Compounds

Rizwana Dilshad[1], Kashif-ur-Rehman Khan[1*], Saeed Ahmad[1], Maqsood Ahmed[1], Huma Rao[1], M Yasmin Begum[2]*

[1]Department of Pharmaceutical Chemistry, Faculty of Pharmacy, The Islamia University of Bahawalpur, Bahawalpur, Pakistan

[2]Department of Pharmaceutics, College of Pharmacy, King Khalid University, Abha, Saudi Arabia

*** Correspondence:**
Kashif-ur-Rehman Khan
kashifur.rahman@iub.edu.pk.

M Yasmin Begum

1 Supplementary Data

Not applicable.

2 Supplementary Figures and Tables

Figure of RP-UHPLC-MS including TIC chromatograms and detailed peaks matching the library of compounds is provided as supplementary material in the following section (may be included in final manuscript if necessary).

2.1 Supplementary Figures

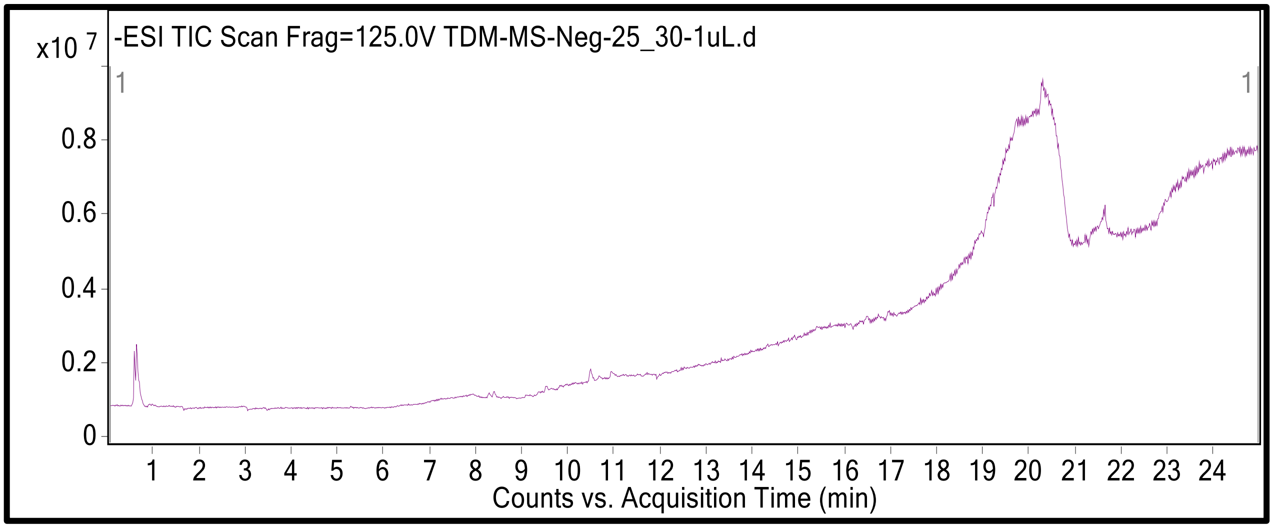

Supplementary Figure 1. Chromatographic representation (TIC) of TDME by using UHPLC-Q-TOF-MS (-ve mode). .

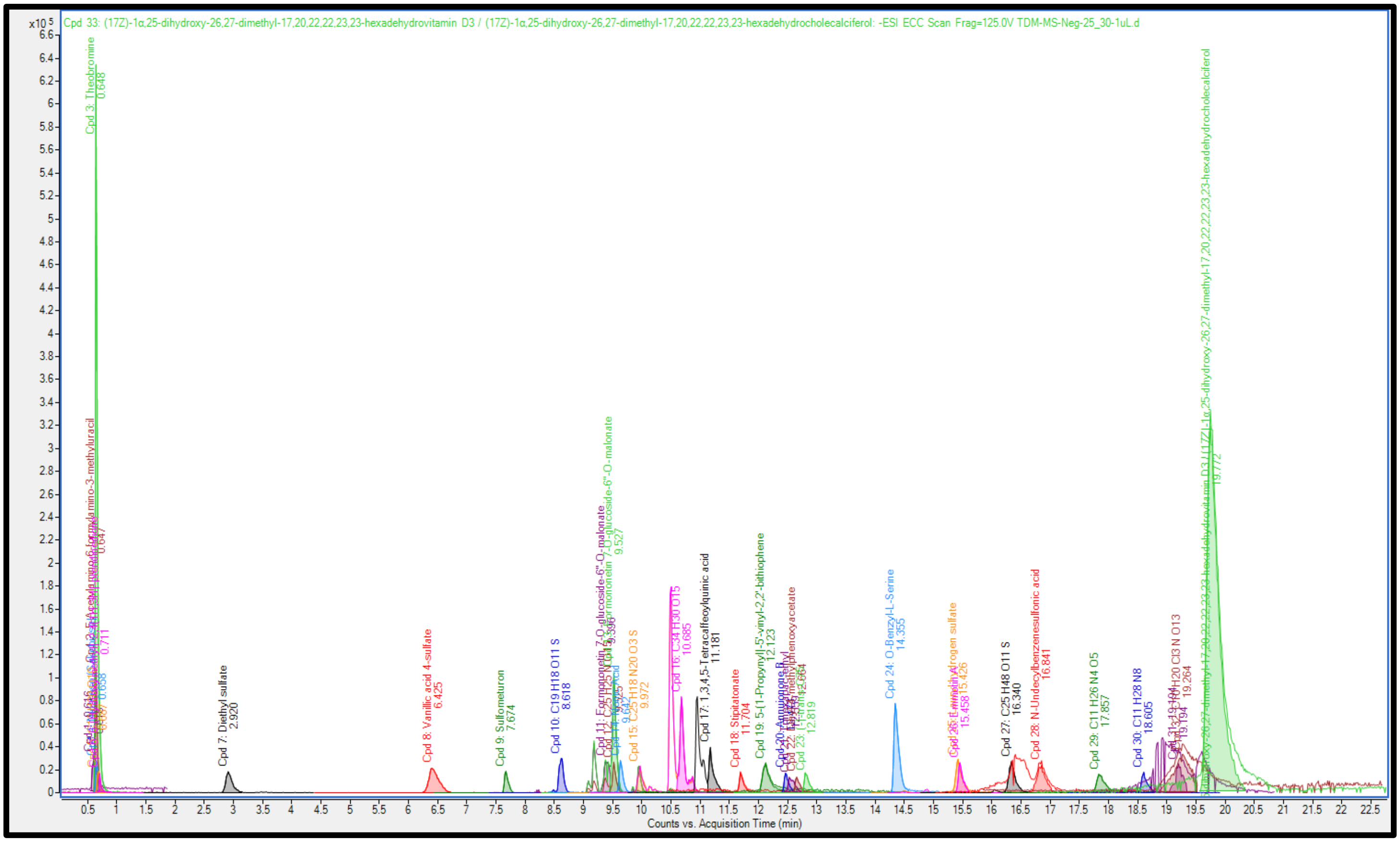

Supplementary Figure 2. Chromatographic representation (with library) of TDME by using UHPLC-Q-TOF-MS (-ve mode).

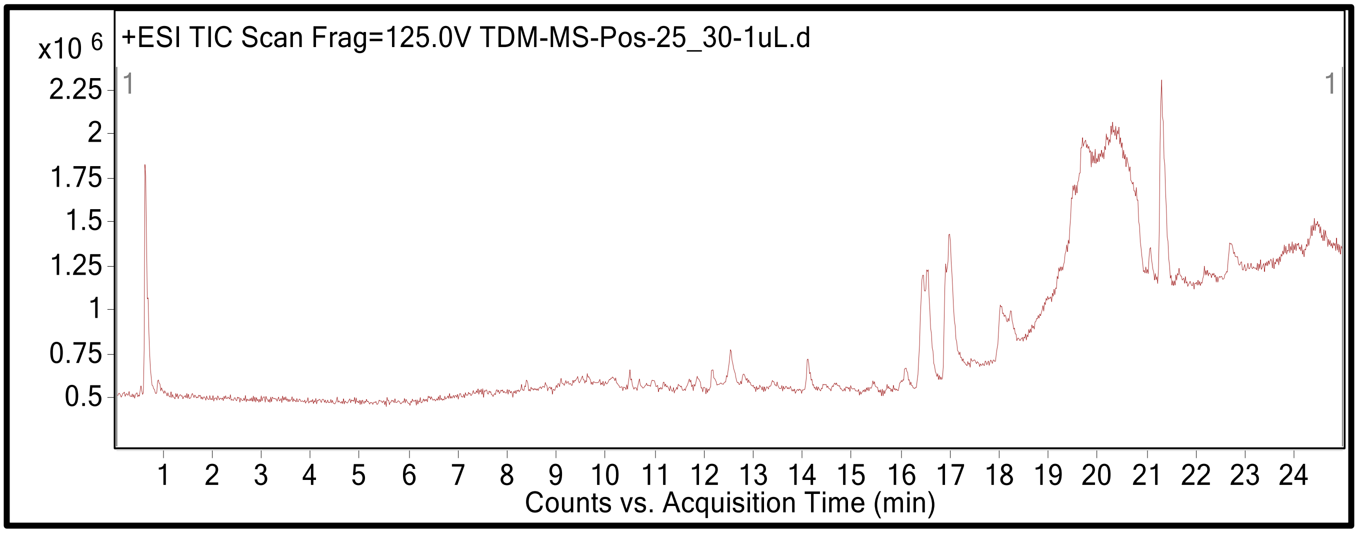

Supplementary Figure 3. Chromatographic representation (TIC) of TDME by using UHPLC-Q-TOF-MS (+ve mode),

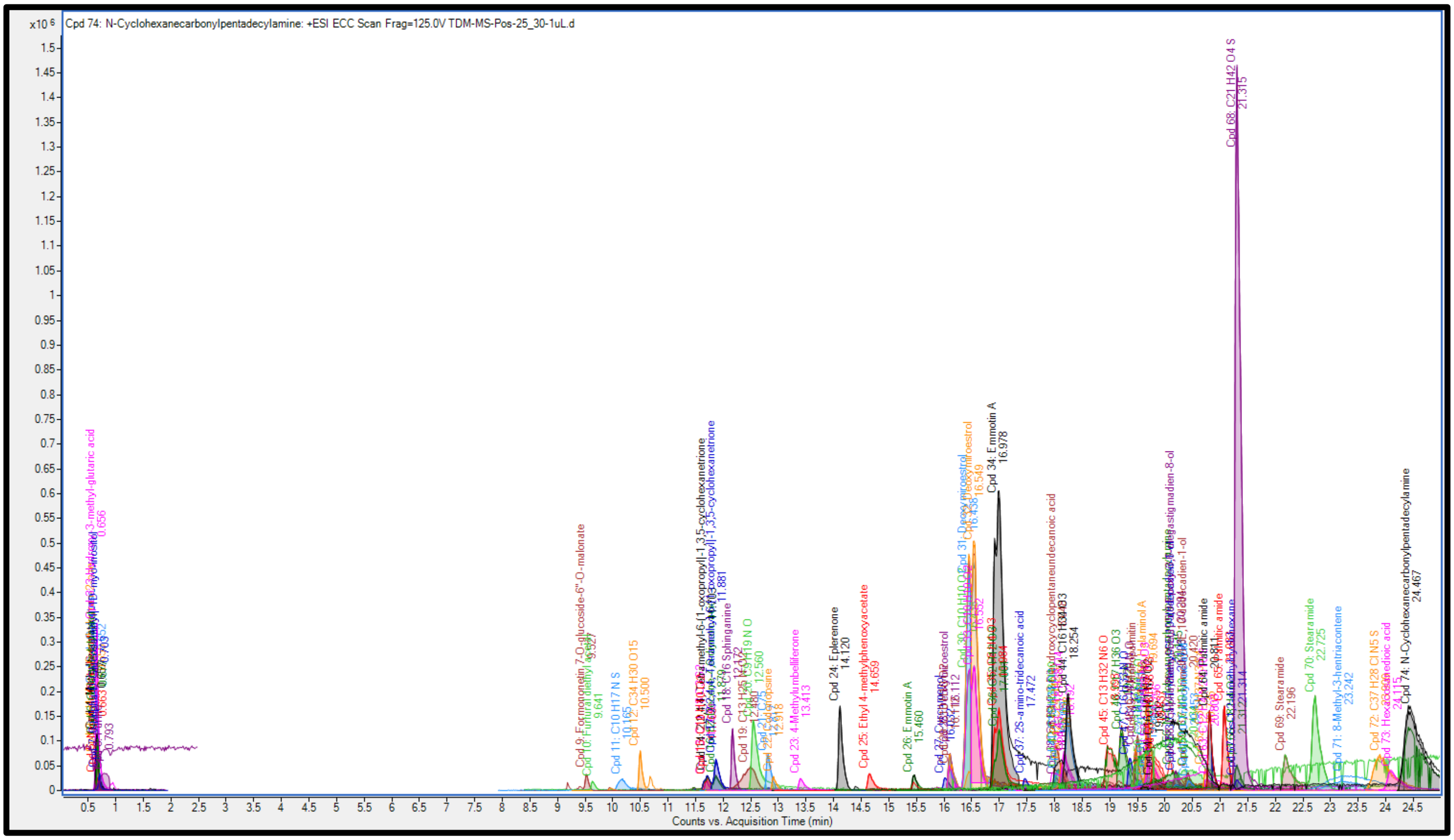

Supplementary Figure 4. Chromatographic representation (with library) of TDME by using UHPLC-Q-TOF-MS (+ve mode).
